# Exchange Characteristics in Interprofessional Collaboration Between Clinical Pharmacists and Physicians: A National Survey

**DOI:** 10.3390/healthcare14182904

**Published:** 2026-09-08

**Authors:** Zikang Yang, Chuchuan Wan, Xiaoyu Xi, Yuankai Huang

**Affiliations:** The Research Center of National Drug Policy & Ecosystem, China Pharmaceutical University, Nanjing 211100, China; yangzikang@stu.cpu.edu.cn (Z.Y.); wanchuchuan@163.com (C.W.)

**Keywords:** clinical pharmacists, physicians, interprofessional collaboration, exchange characteristics, healthcare communication, cross-sectional study

## Abstract

**Objectives:** Exchange characteristics describe and evaluate the state of collaboration and the collaborative counterpart from the perspectives of both parties. They reflect the interaction tendencies between collaborators and are important factors influencing the effectiveness of interprofessional teamwork. This study aimed to assess the current exchange characteristics between clinical pharmacists and physicians and identify between-group differences. It also explored approaches to enhancing professional respect, trust, and clarity of Role Recognition among physicians and clinical pharmacists. **Method:** Separate questionnaire versions were developed for clinical pharmacists and physicians, and a nationwide cross-sectional survey was conducted among both groups. First, descriptive statistics were used to characterize the current status of exchange characteristics in interprofessional collaboration. Second, paired-samples t-tests were conducted to compare clinical pharmacists and physicians across 774 matched pairs. Finally, subgroup analyses were performed using data from 448 clinical pharmacist questionnaires and 446 physician questionnaires. Kruskal–Wallis tests, Mann–Whitney U tests, and chi-square tests were used to examine differences in exchange characteristics across healthcare professionals with different characteristics. **Results:** A total of 448 clinical pharmacist and 446 physician questionnaires were analyzed. Compared with clinical pharmacists, physicians reported greater professional respect (*p* < 0.001), clearer role recognition (*p* < 0.001), better team communication ability (*p* < 0.001), and a stronger sense of fairness (*p* < 0.001) in interprofessional collaboration. In subgroup analyses, clinical pharmacists who graduated from universities and those with higher educational attainment reported greater professional respect. Younger clinical pharmacists reported lower levels of fairness and trust, while physicians in central regions and those in non-permanent employment experienced more severe job burnout. **Conclusions:** Exchange characteristics differed significantly across healthcare professionals by training pathway, region, hospital grade, employment status, gender, age, and education. To strengthen interprofessional collaboration, efforts should focus on optimizing training models, enhancing early-career professional education, improving remuneration systems, and clarifying role boundaries.

## 1. Introduction

Effective pharmacotherapy benefits from minimizing medication-related risks [1]. However, as diseases become increasingly complex and therapeutic options continue to diversify, ensuring medication safety has emerged as a global challenge. Evidence suggests that the effective management of adverse drug reactions is closely associated with the quality of communication between clinical pharmacists and physicians [2]. Deliberate and structured communication and collaboration between these professionals not only facilitates the design of complex medication regimens but also contributes to improved health outcomes [3,4]. In this context, building strong collaborative relationships is both clinically meaningful and organizationally valuable.

Existing research classifies factors influencing collaborative working relationships between physicians and clinical pharmacists into three categories: individual characteristics, context characteristics, and exchange characteristics. Exchange characteristics refer to the psychological tendencies manifested by both parties during collaborative work and are distinct from individual characteristics, such as age and professional background, and context characteristics, such as institutional arrangements and resources. Exchange characteristics dynamically reflect the quality and depth of collaboration. They include interest in collaboration, trust in and professional respect for collaborative partners, communication ability, understanding of the roles and functions of each party in the collaborative process, and expectations regarding collaborative outcomes. Unlike concepts such as work support and job satisfaction examined in previous studies, exchange characteristics emphasize the perspectives of both collaborators. They directly reflect the state of collaboration through evaluations of the collaborative partner and the collaborative relationship. Compared with behavioral or contextual concepts such as team climate and interprofessional collaboration, exchange characteristics focus more specifically on the interpersonal relationship between particular collaborators within a team. McDonough and Doucette (2004) [5] pointed out that in the collaborative working relationship (CWR) model, exchange characteristics show the highest correlation with the core dimensions used to measure collaboration levels: relationship initiation, trustworthiness, and Role specification. Positive exchanges enable clinical pharmacists to establish a CWR with physicians more easily. Such exchanges also help pharmacists gain physician trust to facilitate information flow and clear up Role specification to enhance team harmony [6]. Furthermore, research by Doucette and Nevins confirmed that the value of exchange characteristics, such as trust and role awareness, strengthens as the level of collaboration increases [7]. However, establishing positive exchange characteristics between clinical pharmacists and physicians still faces multiple challenges in China. Role specification remains particularly ambiguous for clinical pharmacists in secondary hospitals and the central and western regions [8,9]. Additionally, physicians show relatively low professional trust in pharmacists [10], and younger clinical pharmacists perceive a lower sense of equity during collaboration [11]. Clinical pharmacists, especially female ones, also face insufficient professional respect [12,13]. Compounded by a generally low frequency of interaction, these issues significantly impair the overall effectiveness of collaboration.

Existing studies have focused on improving exchange characteristics to enhance collaboration within interprofessional teams. In the U.S. healthcare system [14], mutual understanding, trust, bridged communication gaps, and clear collaborative responsibilities serve as the foundation for effective interprofessional cooperation. Similarly, practical evidence from Poland [15] confirms that mutual respect, trust, and strong communication between clinical pharmacists and physicians significantly improve clinical collaboration. Chinese researchers [16] have also verified the significant value of exchange characteristics. However, existing research has largely focused on a limited number of characteristics, such as respect, trust, and Role Recognition. Corresponding recommendations have generally remained at a broad level, such as “strengthening communication” and “maintaining respect.” Detailed analyses of other exchange characteristics, including frequency of collaboration, Fairness, and collaborative attitudes, remain limited. Differences in exchange characteristics across sex, age groups, hospital levels, and regions have also received insufficient attention. Accordingly, this study aimed to comprehensively investigate the current status of exchange characteristics between physicians and clinical pharmacists. More specific exchange characteristics, such as collaborative attitudes and frequency of collaboration, were incorporated into the analysis to provide insights for refining and updating the exchange characteristics in the CWR framework. In addition, differences across population groups were compared to identify groups experiencing difficulties in the exchange process. The findings are expected to inform targeted interventions by healthcare management authorities and facilitate the better integration of clinical pharmacists into healthcare teams.

## 2. Materials and Methods

### 2.1. Research Design

This study was conducted in parallel with a separate investigation focusing on the factors influencing the integration of clinical pharmacists into multidisciplinary care teams [16]. That parallel review systematically synthesized existing literature on the determinants of physician–pharmacist collaboration and assessed their practical relevance within the context of China’s healthcare system. Building upon those findings, the present study narrowed its focus to exchange characteristics, which have the most significant impact on collaboration effectiveness, and analyzed the same dataset. Data collection took place from 1 July to 31 August 2022, using a nationwide, cross-sectional questionnaire survey. Because the distribution of medical resources in China varies across provinces and regions, this study employed a multi-stage stratified sampling method to survey 31 provinces, autonomous regions, and municipalities across mainland China. The sampling process was conducted as follows. Based on per capita disposable income in 2021, all provinces (autonomous regions and municipalities) in China were divided into three groups: high-, middle-, and low-income regions. The number of hospitals planned to be included in each stratum was allocated according to the proportion of hospitals in that stratum relative to the total number of hospitals nationwide, based on the annual health statistics yearbook 2021. At least two clinical pharmacist questionnaires and their corresponding paired physician questionnaires were collected from each sampled hospital to ensure that the sample structure was consistent with the overall distribution of healthcare resources in China. Because clinical pharmacists and physicians participating in the survey were required to have clinical collaboration experience, convenience sampling was used to select voluntarily participating hospitals across different levels. In addition, snowball sampling was applied: after completing the questionnaire, clinical pharmacists were asked to recommend physicians with whom they had collaborative work experience to complete the corresponding questionnaire. During sampling, different participants could recommend the same collaborative partner. However, as long as each pairing relationship was unique and non-duplicated, it was considered a valid matched pair. This study used a national multicenter sample, and the response rates for each sampling stratum are presented in Table 1.

The sample size was calculated as follows. The significance level was set at α = 0.05 (two-sided), with a statistical power of 0.90 (β = 0.10). The expected paired mean difference (Δ) and the standard deviation of the paired differences (σ_d_) were estimated from pilot study data. Because hospital-level clustering could be present, the design effect (DEFF) was calculated using the intraclass correlation coefficient (ICC) obtained from the pilot study and the average number of matched pairs recruited per hospital (m). The required sample size was then estimated using the paired-sample size formulas shown in Equations (1) and (2):

n = [(Z_1−α/2_ + Z_1-β_)2σ_d_^2^/Δ^2^] × DEEF (1)

DEFF = 1 + (m − 1) × ICC(2)

The calculation indicated that a minimum of 92 matched pairs was required per group. Considering that stratified sampling was used in this study with three strata and that some differences were expected across strata, the target sample size was further adjusted. In addition, an approximately 50% sample exclusion rate reported in similar studies [16] was taken into account. Accordingly, the final planned sample size was 564 matched pairs. A total of 448 clinical pharmacist questionnaires and 446 physician questionnaires were ultimately included. After matching, 774 valid matched pairs were identified, which met and exceeded the required sample size.

### 2.2. Instruments

To comprehensively summarize the dimensions of exchange characteristics, this study adopted the collaborative working relationship (CWR) model proposed by McDonough and Doucette [17] (2005) as its theoretical framework. This model provides a well-established classification of influencing factors and is relatively well suited to the Chinese context. It classifies factors influencing collaboration between physicians and clinical pharmacists into three categories: individual characteristics, context characteristics, and exchange characteristics. Building on this framework, we systematically reviewed the Chinese and international literature to identify various factors associated with collaboration and further screened those related to exchange characteristics. Subsequently, through expert consultation, conceptually similar elements within the exchange characteristics were integrated, indicator names were standardized, and their conceptual descriptions were refined to improve their appropriateness. Ultimately, 14 exchange characteristics, including respect and trust, were identified.

A self-developed questionnaire was used in this study. The first section collected demographic information, including respondents’ age, gender, professional title, and educational attainment, consistent with the parallel study conducted on the integration of clinical pharmacists into multidisciplinary care teams. The selection of exchange characteristics variables was informed by existing literature [18,19] (see Table 2a) and conceptually aligned with those used in the parallel study.

A total of 14 exchange characteristics were included in the questionnaire: professional respect, trust, role recognition, perceived fairness, job burnout, perceived usefulness, collaborative expectations, team consistency, frequency of collaboration, outside-of-work socialization, team communication ability, general language competence, power influence, and non-power influence (the paraphrases are shown in Table 2a). These characteristics were measured using 47 items. Except for the job burnout subdimension, which was reverse-scored under the broader construct of collaborative attitudes, higher scores indicated more favorable levels of each characteristic (item examples are provided in Table 2b).

Two versions of the questionnaire were developed: a physician version and a clinical pharmacist version. Physicians completed the physician version, while clinical pharmacists completed the pharmacist version. The main difference between the two versions lies in their perspectives. The physician questionnaire assesses physicians’ perceptions of exchange characteristics in collaboration with clinical pharmacists (e.g., “Do you feel respected by clinical pharmacists?”). In contrast, the pharmacist questionnaire examines clinical pharmacists’ perceptions of similar exchange characteristics when collaborating with physicians (e.g., “Do you feel respected by physicians?”).

The reliability analysis showed that the overall Cronbach’s α coefficient was 0.95 for the clinical pharmacist version of the questionnaire and 0.98 for the physician version. The Cronbach’s α coefficient for each dimension exceeded 0.70 (see Tables S15 and S16 in Supplement S2). Confirmatory factor analysis (CFA) showed that the composite reliability (CR) for each dimension exceeded 0.70, and the average variance extracted (AVE) exceeded 0.50 (see Table S17 in Supplement S2). These findings indicate that both versions demonstrated good internal consistency, convergent validity, and a satisfactory factor structure. In addition, measurement invariance was assessed using multigroup confirmatory factor analysis (MG-CFA) (see Table S18 in Supplement S2). From weak invariance (M_2_) to strong invariance (M_3_), the changes in model fit were ΔCFI = −0.008 and ΔRMSEA = 0.001. Both met the recommended cutoff criteria proposed by Chen (2007) [20] (ΔCFI ≥ −0.010 and ΔRMSEA ≤ 0.015), indicating measurement invariance across the clinical pharmacist and physician versions of the questionnaire.

### 2.3. Data Analysis

After training, more than 400 undergraduate students from schools of pharmacy were recruited as survey investigators. Investigators conducted face-to-face surveys using mobile devices. After obtaining respondents’ consent, they explained the nature, purpose, requirements, and relevant precautions of the survey. The questionnaires were administered anonymously, and no personally identifiable information was collected. All data were stored on the device of the principal investigator. To ensure the authenticity and reliability of the research process and findings, two data reviewers were appointed. They conducted preliminary checks of the appropriateness of the research procedures and the timeliness and completeness of questionnaire completion. Only the reviewers and the principal investigator were authorized to access the raw data.

**Figure 1:**
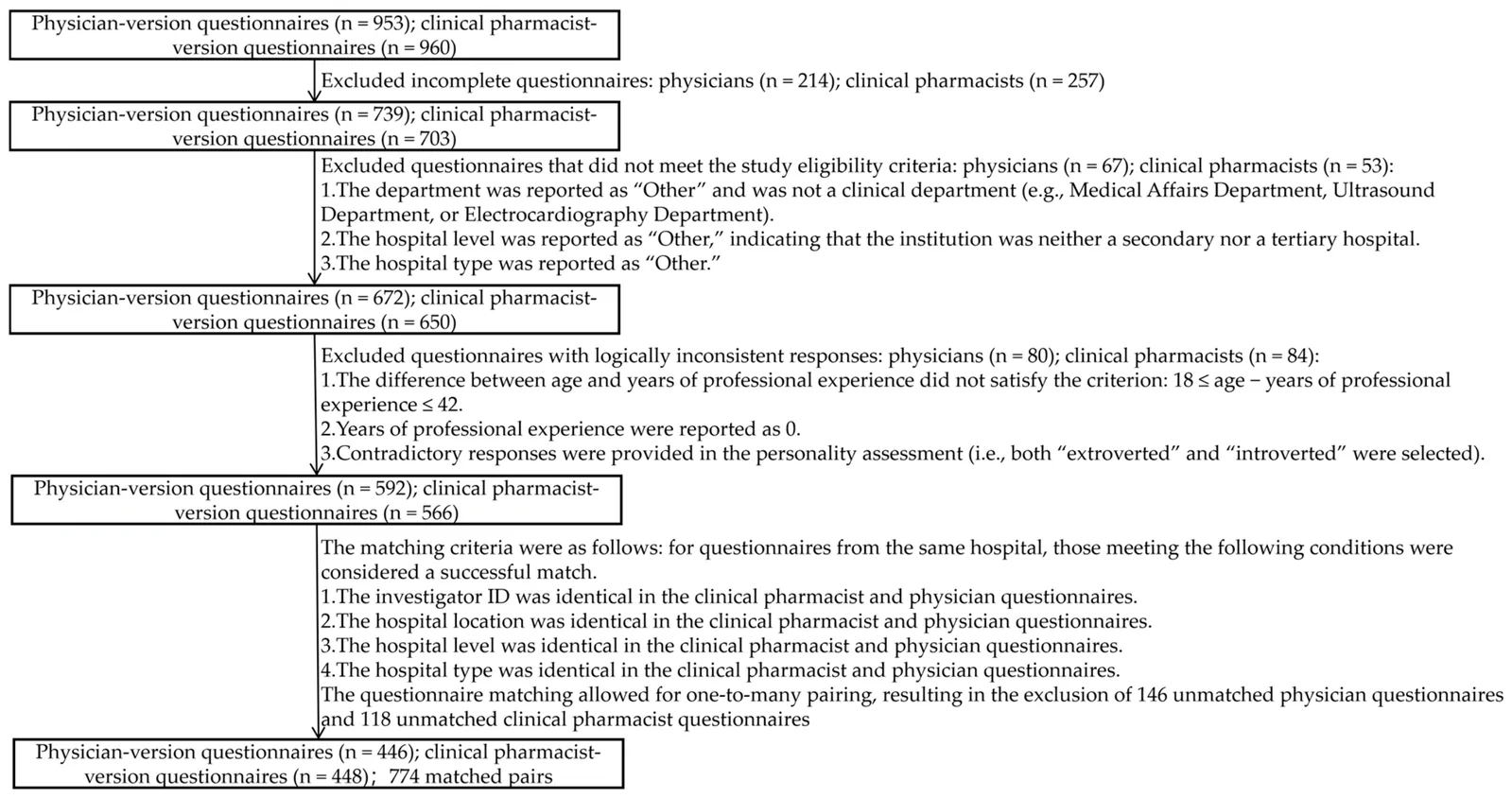
Questionnaire Cleaning and Matching Process.

In this study, the raw data underwent a rigorous process of screening, organization, and analysis. Data cleaning was conducted in two phases. First, questionnaires were scrutinized based on specific exclusion criteria to identify invalid entries. The exclusion criteria included: (1) respondents who did not meet the enrollment requirements, such as physicians and pharmacists not directly involved in clinical treatment or those not working in secondary or tertiary hospitals; (2) incomplete questionnaires; or (3) unrealistic responses or logical contradictions, such as reporting zero years of professional experience or exhibiting contradictory traits in personality tests. Second, questionnaire matching was performed. The matching process for valid questionnaires was conducted as follows. For clinical pharmacist and physician questionnaires from the same hospital, four items were combined to generate a unique identifier: investigator ID, region (province, city, and district), hospital level (secondary or tertiary), and hospital type (rehabilitation, integrated traditional Chinese and Western medicine, traditional Chinese medicine, specialized, or general hospital). The identifiers generated from the clinical pharmacist and physician questionnaires were then compared. If a clinical pharmacist’s identifier exactly matched an identifier in the physician questionnaires, or vice versa, the clinical pharmacist and physician were considered to have had collaborative experience, and the questionnaires were regarded as successfully matched. A one-to-many matching approach was adopted, whereby one clinical pharmacist or physician could be matched with multiple physicians or clinical pharmacists, respectively, to form collaborative pairs. For participants from the same hospital, a questionnaire was considered eligible for matching as long as a corresponding collaborator questionnaire could be identified. Therefore, the same questionnaire could be included in multiple matched pairs. As a result, the final number of matched pairs (774 pairs) did not correspond exactly to the numbers of unique participants (448 clinical pharmacists and 446 physicians). Questionnaires that could not be successfully matched were excluded, even if they were otherwise valid. The questionnaire cleaning and matching process is presented in Figure 1.

After data cleaning, descriptive statistics were performed for responses to both versions of the questionnaire, covering all exchange characteristics and their subdimensions, to characterize the current status of exchange characteristics between clinical pharmacists and physicians. In addition, based on the 774 matched pairs, paired-samples t-tests were conducted to compare differences in exchange characteristics between physicians and clinical pharmacists.

Given that this study did not focus on the causal relationship between exchange characteristics and the level of collaboration, but rather on differences in exchange characteristics across healthcare professional groups, further subgroup analyses were conducted for clinical pharmacists and physicians. Between-group comparisons were then performed to identify group-specific differences in exchange characteristics within interprofessional collaboration. Subgroup analysis took into account sociodemographic factors that notably influence the level of cooperation, including clinical pharmacists’ training methods (i.e., training after graduation, training after job transfer, direct assignment without training), hospital grades (i.e., secondary hospital, tertiary hospital), regional locations (i.e., eastern, central, western), and physicians’ employment forms (i.e., regular vs. non-regular employment) [16]. Additionally, the literature indicated notable variations in occupational health related to gender, age, and education, suggesting a conceptual link between occupational health and exchange characteristics among healthcare professionals [11,21,22]. The study anticipated identifying statistically significant differences in exchange characteristics across these subgroups. After subgroup classification, normality was first assessed for the data within each subgroup. One-way analysis of variance (ANOVA) was used for subgroups with normally distributed data. For non-normally distributed data, the Mann–Whitney U test was used for comparisons between two groups, whereas the Kruskal–Wallis test was used for comparisons involving more than two groups. Subgroup analyses were conducted using the actual sample sizes of 448 clinical pharmacists and 446 physicians to identify within-group differences. To control Type I error arising from multiple comparisons, Bonferroni correction was applied to all subgroup analyses involving one-way ANOVA or Kruskal–Wallis tests, including regional locations and clinical pharmacists’ training methods. The adjusted *p* values were calculated by multiplying the original *p* values by the number of pairwise comparisons (m), which was calculated using Equation (3), where n represents the number of groups compared.

m = [n × (n − 1)]/2(3)

In addition, using the hospital as the clustering unit, a linear mixed-effects model was applied to estimate the within-hospital variance (σ_W_^2^) and between-hospital variance (σ_B_^2^). These estimates were then entered into Equation (4) to calculate the intraclass correlation coefficient (ICC), thereby assessing the hospital-level clustering effect in the sample. The results of the generalized estimating equations (GEE) analysis were also reported to re-evaluate the main findings while accounting for hospital-level clustering.

ICC = σ_B_^2^/(σ_W_^2^ + σ_B_^2^) (4)

## 3. Results

A total of 960 clinical pharmacist questionnaires and 953 physician questionnaires were collected. After data cleaning and the exclusion of questionnaires with incomplete or implausible responses, 566 valid clinical pharmacist questionnaires and 592 valid physician questionnaires remained, resulting in 774 valid matched pairs. The sample covered 275 hospitals across all 31 provinces, autonomous regions, and municipalities in China. As shown in Table 3a, the institutional distribution was broadly consistent with data from the Annual Health Statistics Yearbook 2021, indicating good geographic coverage at the institutional level and suggesting reasonable sample representativeness.

### 3.1. Demographic Information

Among clinical pharmacists, women comprised a substantial proportion at 58.7%, whereas the gender distribution among physicians was relatively balanced, with women making up 49.33% of the cohort. The average age of clinical pharmacists was 38.46 ± 7.25 years, compared to 42.23 ± 7.88 years for physicians, with the majority of both groups falling within the 35-to-44-year age range. In terms of marital status, the majority of participants were married. Clinical pharmacists had an average length of service of 10.88 ± 6.67 years, whereas physicians had a longer average length of service at 15.22 ± 7.61 years. Both clinical pharmacists (38.2%) and physicians (27.1%) predominantly worked in internal medicine. Detailed demographic information is provided in Table 3b.

### 3.2. Exchange Characteristics

The results showed that, among the 774 matched pairs, physicians generally had higher scores than clinical pharmacists for continuous measures of exchange characteristics. Although hospital-level clustering was present, the generalized estimating equation (GEE) results indicated that clustering had only a limited impact on statistical significance (see Table 4 for details). The main text reports only the results that remained statistically significant after adjustment, while the remaining results are provided in Supplement S1: Subgroup Analysis Results.

#### 3.2.1. Respect

**Figure 2:**
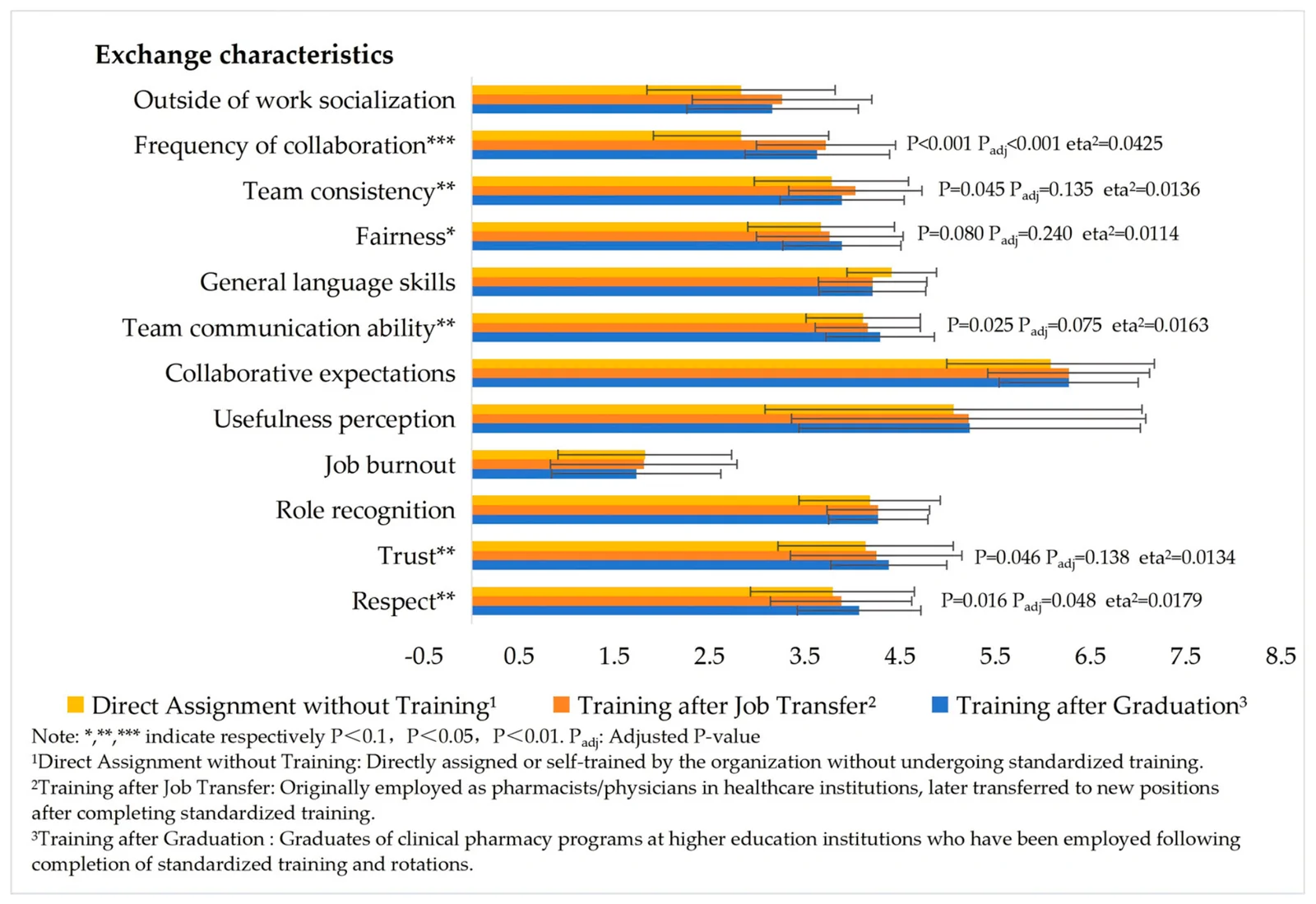
Differences in Exchange Characteristics Among Clinical Pharmacists Across Training Pathways.

**Figure 3:**
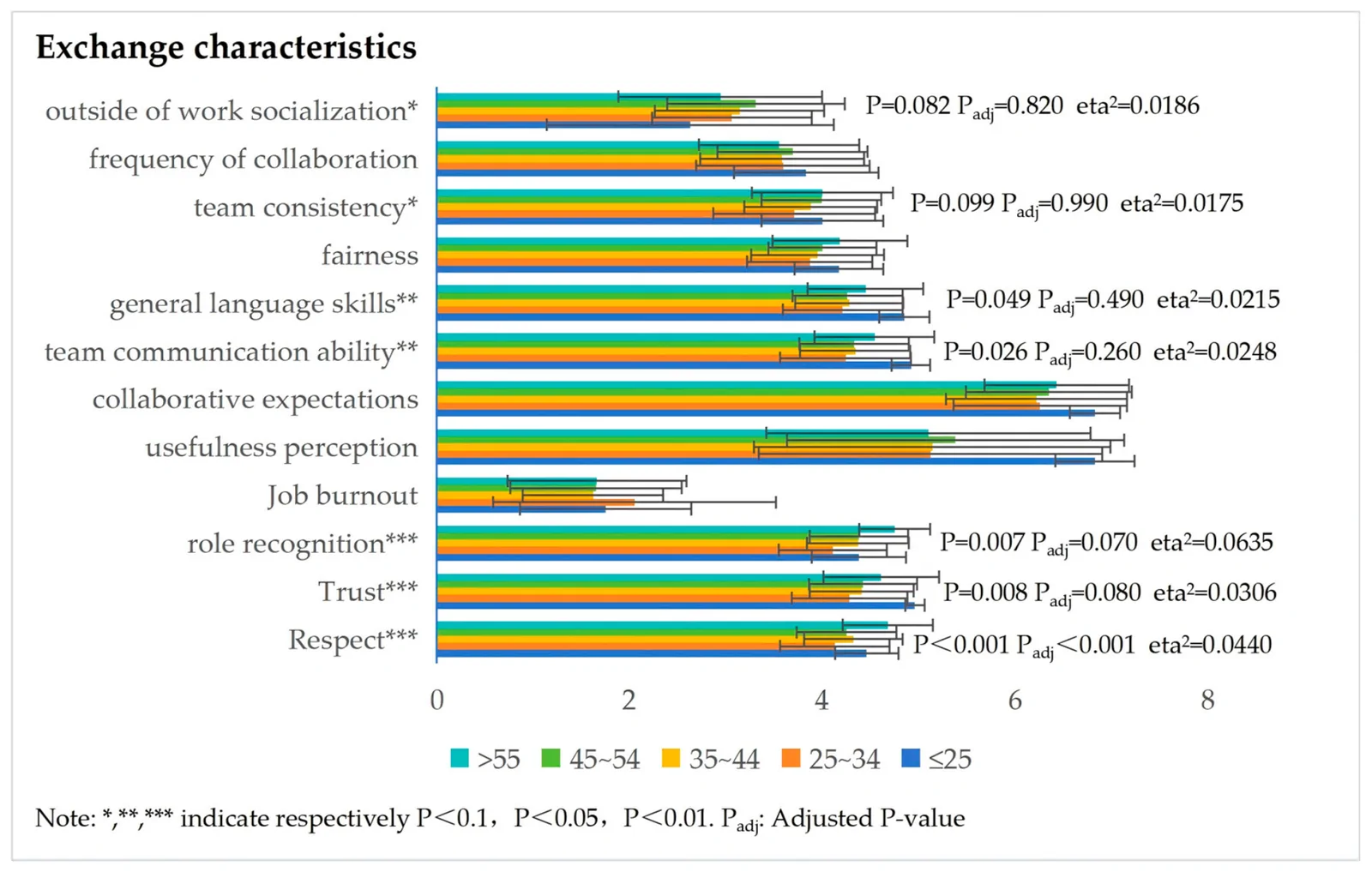
Differences in Exchange Characteristics Among Physicians by Age Group.

Clinical pharmacists reported a lower level of Respect compared to physicians. Among clinical pharmacists, those who graduated from higher education institutions achieved higher scores in the Respect dimension (*p* < 0.05; Figure 2). Within the physician group, older physicians (*p* < 0.01; Figure 3) scored higher in the Respect dimension.

#### 3.2.2. Trust

There was no significant difference in trust between physicians and clinical pharmacists. Among clinical pharmacists, those who graduated from higher education institutions achieved higher scores in the Trust dimension (*p* < 0.05; Figure 2). Within the physician group, younger physicians scored higher in the Trust dimension (*p* < 0.01; Figure 3).

#### 3.2.3. Role Recognition

Clinical pharmacists achieved lower scores in the role recognition dimension compared to physicians. Within the physician group, older physicians scored higher in role recognition (*p* < 0.01; Figure 3).

#### 3.2.4. Fairness

**Figure 4:**
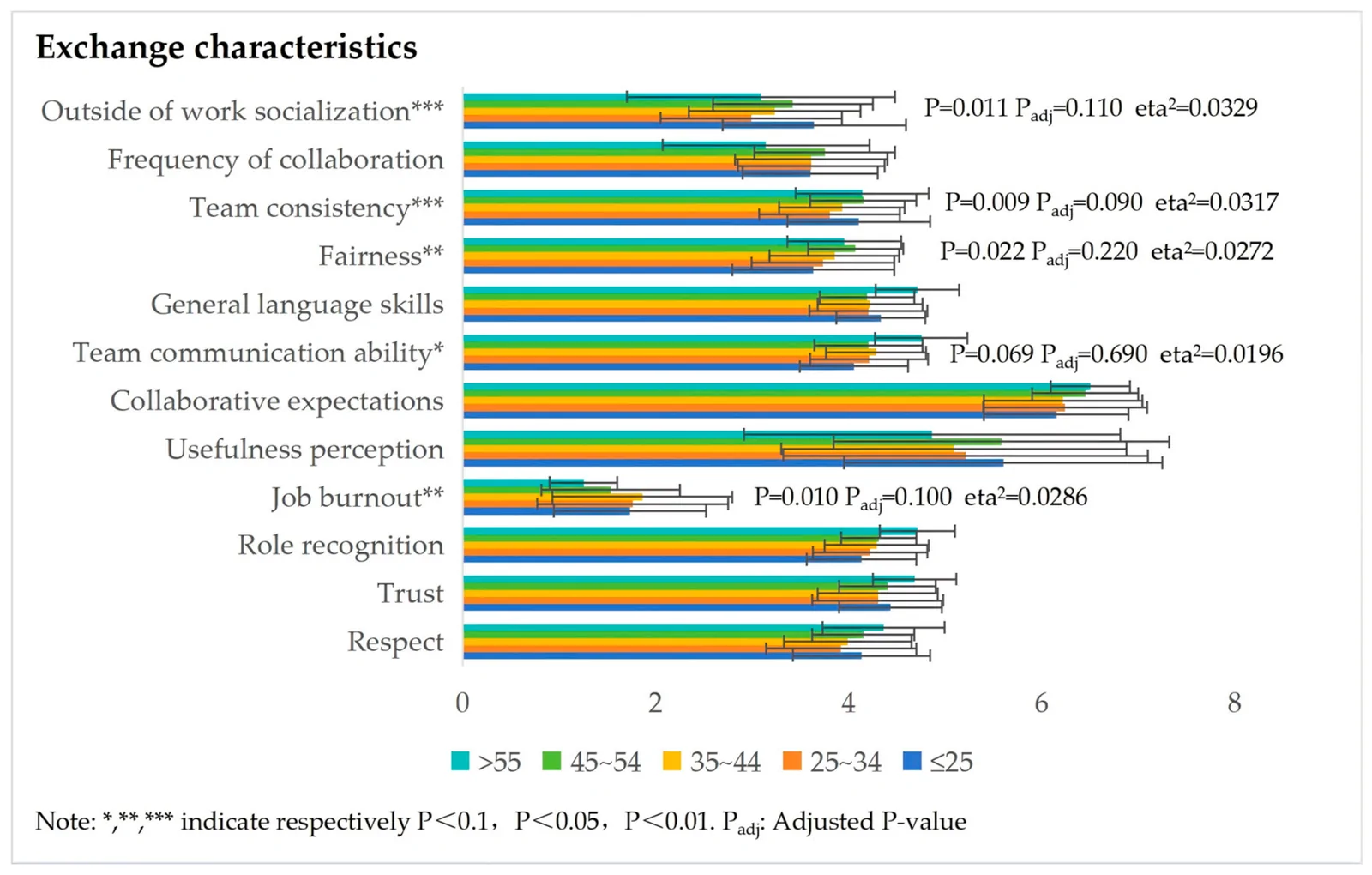
Differences in Exchange Characteristics Among Clinical Pharmacists by Age Group.

Physicians scored higher in the Fairness dimension compared to clinical pharmacists. Within the clinical pharmacist group, older clinical pharmacists reported a higher sense of perceived fairness (*p* < 0.05; Figure 4).

#### 3.2.5. Job Burnout

**Figure 5:**
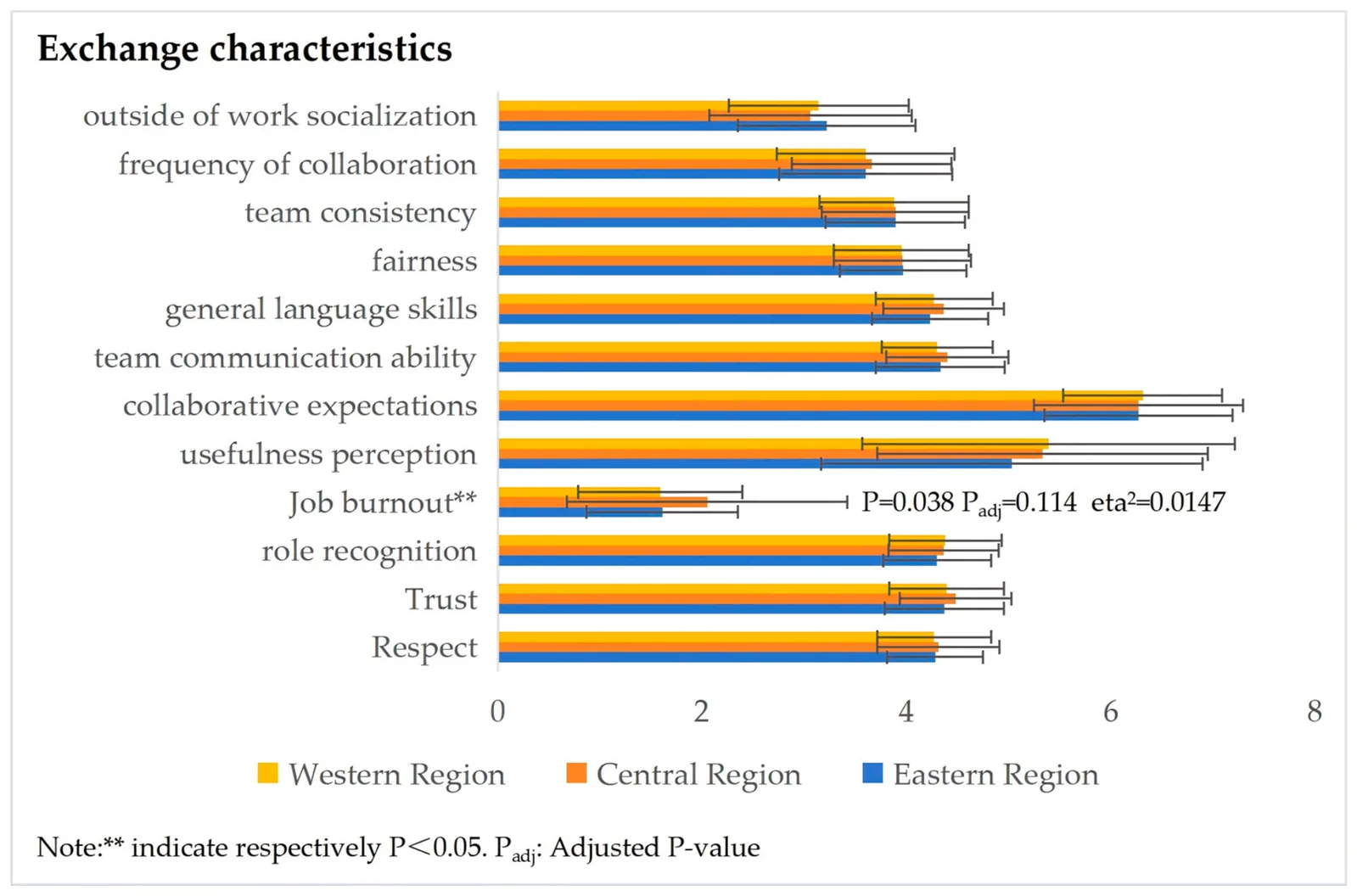
Differences in Exchange Characteristics Among Physicians by Region.

**Figure 6:**
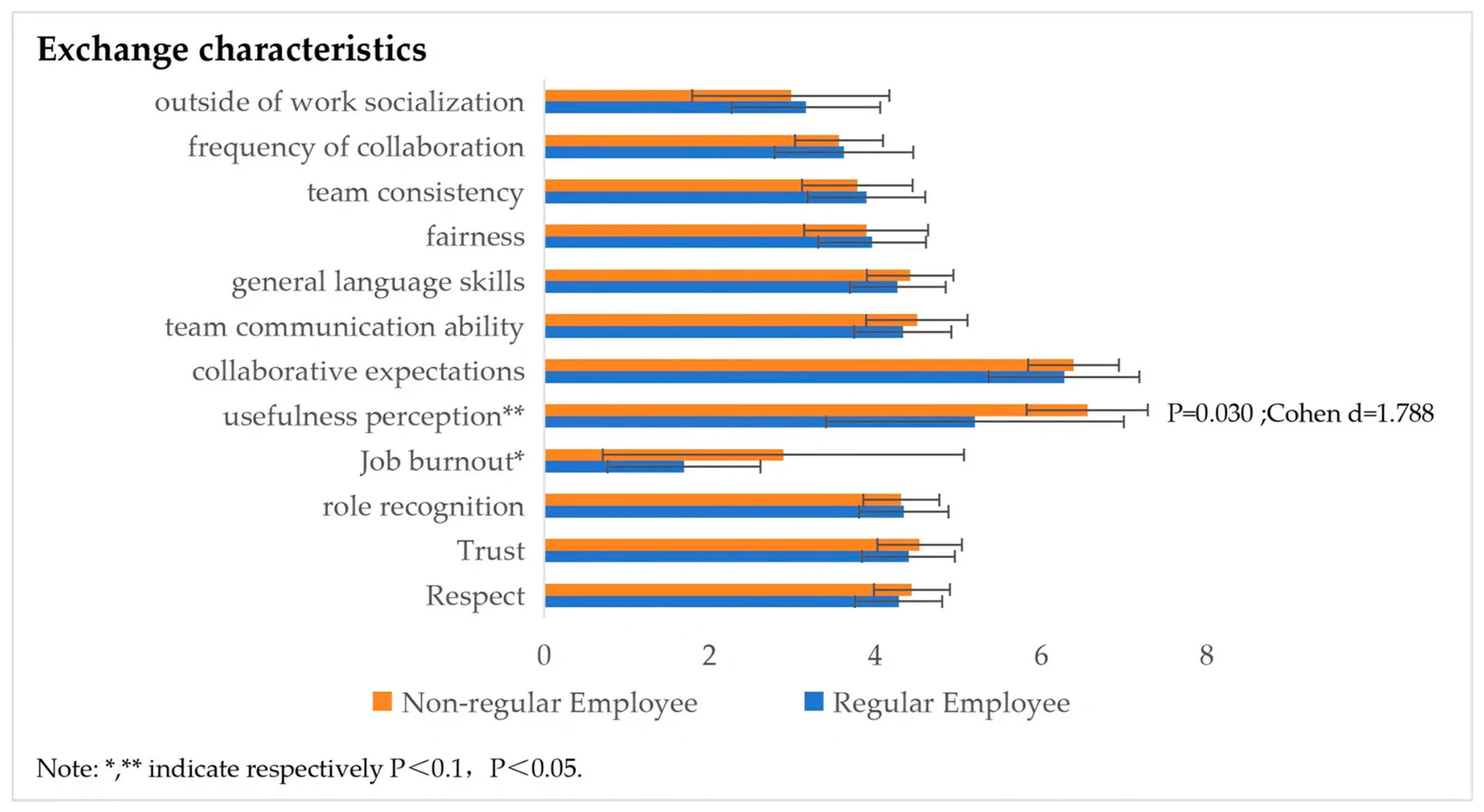
Differences in Exchange Characteristics Among Physicians by Employment Status.

No significant difference in Job Burnout was observed between physicians and clinical pharmacists. Within the physician group, however, those from the central region experienced more severe Job Burnout (*p* < 0.05; Figure 5). Furthermore, non-permanently employed physicians reported marginally more severe Job Burnout (*p* < 0.01; Figure 6).

#### 3.2.6. Usefulness Perception

There was no significant difference in usefulness perception between physicians and clinical pharmacists. Compared to regular employees, non-regular employee physicians demonstrated greater recognition of the usefulness of interdisciplinary clinical collaboration (*p* < 0.05, as shown in Figure 6).

#### 3.2.7. Team Consistency

No significant difference in Team Consistency was observed between physicians and clinical pharmacists. However, among clinical pharmacists, those who underwent training after job transfer or training after graduation achieved higher scores in Team Consistency compared to those from other training pathways (*p* < 0.05; Figure 2).

#### 3.2.8. Outside of Work Socialization

No significant difference in Outside of Work Socialization was observed between physicians and clinical pharmacists. However, among clinical pharmacists, younger clinical pharmacists demonstrated a higher willingness to engage in Outside of Work Socialization with physicians (*p* < 0.05; Figure 4).

#### 3.2.9. Frequency of Collaboration

There was no significant difference in frequency of collaboration between physicians and clinical pharmacists. Clinical pharmacists who received training after graduation and job transfer demonstrated higher frequency of collaboration compared to those from other training pathways (*p* < 0.01, as shown in Figure 2).

#### 3.2.10. Team Communication Ability

Physicians demonstrated higher team communication ability than clinical pharmacists. Within the physician group, younger physicians showed stronger team communication ability compared to older physicians (*p* < 0.05, as shown in Figure 3). Among clinical pharmacists, those who received training after graduation exhibited superior team communication ability compared to clinical pharmacists from other training pathways (*p* < 0.05, as shown in Figure 2).

## 4. Discussion

### 4.1. Summary

This study offers a comprehensive evaluation of the current status of exchange characteristics between clinical pharmacists and physicians, and examines the differences in these characteristics across various groups. The findings indicated that physicians scored higher than clinical pharmacists in exchange characteristics. Further analysis revealed significant differences in exchange characteristics based on individual attributes such as gender, age, and educational background. In addition, notable variations in exchange characteristics were observed among physicians from different regions, hospital grades, and employment types, as well as among clinical pharmacists with different training backgrounds. It should be noted that this study used a cross-sectional design and aimed to identify associations between group differences and exchange characteristics rather than establish causal relationships. Future empirical studies are needed to further explore the mechanisms underlying these observed differences.

#### 4.1.1. Occupation

Respect is a key indicator of whether clinical pharmacists and physicians can freely and directly provide or receive feedback during collaboration, and whether clinical pharmacists’ professional autonomy is ensured. In China, clinical pharmacists reported a lower level of respect compared to physicians. Other scholars have also pointed out that physicians continue to dominate clinical decision-making, while clinical pharmacists hold a relatively marginal position in interprofessional treatment teams [23,24].

The development of pharmaceutical care in China started relatively late and still faces several structural barriers, including underdeveloped digital systems, shortages of essential equipment, and insufficient staffing in clinical pharmacy departments—all of which directly hinder clinical pharmacists’ involvement in practice [25,26]. Moreover, clinical pharmacists in China often lack the time to participate in interdisciplinary collaboration. In contrast to the U.S., where pharmacy staff typically provide clinical services for at least eight hours a day [27], only 40% of clinical pharmacists in China’s tertiary hospitals devote 80% or more of their working time to clinical pharmacy-related activities [28]. Additionally, unlike their counterparts in the U.S., Chinese clinical pharmacists do not hold prescribing rights [29] and face policy-level limitations when participating in therapeutic interventions [30,31].

To address these challenges, medical institutions should strictly implement the guidelines set out in the National Health Commission’s 2018 policy document Opinions on Accelerating the High-Quality Development of Pharmaceutical Services. This includes increasing the staffing of pharmaceutical personnel and relieving clinical pharmacists from traditional responsibilities such as medication rounds and dispensing, thereby allowing them to allocate more time to clinical collaboration with physicians. It is also recommended that relevant legal frameworks be expedited and improved to clarify the roles, responsibilities, and rights of clinical pharmacists, thus facilitating their full integration and professional contribution in multidisciplinary clinical care. Within healthcare institutions, interprofessional collaborative teams should be actively established. The American Society of Clinical Oncology (ASCO) advocates a transition from the traditional physician-centered model toward an interdependent multidisciplinary team model [32]. Effective clinical multidisciplinary teams should have clearly defined responsibilities and roles, effective communication, shared goals, and other essential characteristics. Visual tools such as swimlane diagrams could be used to clearly delineate the responsibilities of physicians, clinical pharmacists, nurses, and other healthcare professionals. Team roles and responsibilities should also be regularly reassessed and appropriately allocated to improve collaboration efficiency.

#### 4.1.2. Training Method

Clinical pharmacists who underwent training after graduation or training after job transfer achieved higher scores in respect, team communication skills, and collaboration frequency compared to those under direct assignment without training. By 2019, 52 universities in China offered undergraduate programs in clinical pharmacy [33]. Taking China Pharmaceutical University as an example [34,35], the curriculum integrates foundational theories from medicine, pharmacy, chemistry, and biology. This systematic higher education framework effectively strengthens the professional knowledge base and clinical skills of pharmacists [36,37]. Meanwhile, pharmacists who undergo retraining after transferring into clinical pharmacy roles generally have more extensive prior work experience. Greater clinical experience may be one factor that physicians consider when deciding whether to increase the frequency of collaboration [38]. In turn, this experience may be positively associated with a stronger willingness to collaborate and greater team consistency between physicians and clinical pharmacists.

A joint training mechanism between hospitals and universities could be explored to integrate pharmaceutical theory with clinical practice and enhance the clinical experience of graduates who enter hospitals directly as clinical pharmacists after graduation. This approach may help strengthen physicians’ and clinical pharmacists’ willingness to collaborate. Recent policy documents issued in China stipulate that licensed pharmacists must engage in continuing education to maintain and enhance their pharmaceutical service capabilities after obtaining their professional qualifications. These policies aim to strengthen the management of continuing education for licensed pharmacists and continuously improve their professional competence and pharmaceutical service abilities [39]. In light of these regulations, hospitals should implement incentive and promotion mechanisms to encourage clinical pharmacists with lower academic qualifications, as well as non-college graduates, to engage in ongoing professional development. This approach will enhance their expertise and, ultimately, improve patient care. In addition, the Accreditation Council for Pharmacy Education (ACPE) in the United States has developed standards related to the application of artificial intelligence in pharmacy education [40]. Pharmacy education institutions could draw on this approach and explore the integration of AI technologies into continuing education for clinical pharmacists. For example, AI could be used to simulate clinical communication scenarios, thereby improving clinical pharmacists’ communication skills and facilitating clinical exchange. AI-based scenario simulation could also be used to strengthen collaborative capabilities within interprofessional clinical teams.

#### 4.1.3. Hospital Regional Locations

Previous studies have shown that job burnout among healthcare professionals is closely related to work stress—typically, the longer the working hours and the higher the stress level, the greater the risk of burnout [41]. This study revealed that physicians in central China experienced slightly higher levels of Job Burnout than those in the eastern and western regions. This regional difference may be related to the so-called “central region collapse” effect [42,43,44]. The eastern region benefits from a strong economy and abundant healthcare resources [45], with more efficient systems for resource allocation, resulting in lower stress and less burnout among physicians. The western region, while less economically developed, has received targeted policy support—such as the western development strategy, the “three support and one assistance” program, and county-level medical community reforms—which have led to increased support, including a higher ratio of primary healthcare workers per 1000 people than in the central or eastern regions [46].

In contrast, the central region has struggled to match the eastern region’s resources and has not fully benefited from the preferential policies aimed at the west. These differences in regional policies and resource allocation may partly explain the higher level of Job burnout among healthcare professionals in central China. Future studies could further investigate how regional healthcare resource allocation and targeted support policies may alleviate Job burnout. For example, higher-level governments or regional coordinating bodies could take the lead, with complementary local fiscal support, to increase investment in healthcare resources in less-developed areas. In addition, experience from regional medical alliances and integrated healthcare delivery systems could be used to coordinate healthcare resources more effectively. Promoting the development of regional medical centers may further help reduce inequalities in the geographic distribution of healthcare resources.

#### 4.1.4. Physicians’ Employment Forms

Informal employment generally refers to arrangements such as labor dispatch, temporary employment, and internships, in which employees often occupy relatively lower positions within the workplace hierarchy. Concerns about job security and career continuity may influence their exchange characteristics [47]. However, in this study, informally employed physicians reported higher usefulness perception than formally employed physicians, which did not support this assumption. This finding may be attributable to the very small sample of informally employed physicians (n = 11) and should be verified in larger studies. 

In addition, some findings suggested that informally employed physicians may face a relatively higher risk of job burnout, although this result should be regarded as exploratory. In China, however, informally employed healthcare professionals do face substantial practical challenges: non-regular medical staff often experience workloads comparable to or even greater than those of formally employed staff, while receiving significantly lower salaries and having more limited opportunities for career advancement [48]. Compensation plays a crucial role in fostering effective collaboration [49]. The disparity caused by non-regular status—manifesting as unequal pay for equal work and restricted career advancement—further undermines their perceived organizational support.

According to social exchange theory, a lack of organizational support weakens employees’ emotional attachment to, and sense of responsibility toward, the organization, which in turn negatively affects their work engagement and professional attitude [50]. Similar challenges have been identified in studies from Japan [51] and South Korea [52], where non-regular employees receive lower wages, performance evaluations, benefits, and social security, resulting in greater work-related stress. Future research could further examine the effects of compensation on healthcare professionals’ exchange characteristics. Hospitals may also consider optimizing compensation systems to ensure equal pay rights for employees under different employment arrangements. Social security benefits for informally employed staff should be fully implemented, and equal opportunities for promotion should be provided. These measures may help alleviate work-related stress and Job burnout, thereby improving the working conditions of physicians in informal employment arrangements.

#### 4.1.5. Gender and Age

Previous studies have generally shown that women are more likely to experience discrimination in the workplace [53]. However, the present study revealed a different pattern: physicians of different sexes showed some differences in respect, with female physicians potentially perceiving relatively higher levels of respect. This finding is consistent with evidence from Taiwan [54], where female hospital staff reported significantly more positive perceptions of teamwork climate than their male counterparts. One possible explanation is that female physicians may place greater emphasis on positive relationships when establishing and maintaining collaboration [55], whereas male physicians may exhibit different attitudes for other reasons [56]. In addition, women generally demonstrate stronger team communication ability than men [57]. This advantage may indirectly contribute to female physicians receiving greater respect from their colleagues.

Clinical pharmacists and physicians exhibited differences in several exchange characteristics. The results showed that older clinical pharmacists scored relatively higher in Fairness. This outcome aligns with previous research from Guangdong Province, which showed that younger pharmacists generally feel their salaries fail to match their contributions. Consequently, their perceived organizational fairness remains lower than that of their older colleagues [11]. Additionally, generational effects may also partly explain this difference. Individuals born before the Reform and Opening-up era navigated periods of profound social structural transformation [58]. This shared collective experience could serve as another underlying reason why older clinical pharmacists demonstrate a stronger sense of Fairness. Older clinical pharmacists also performed relatively better in terms of team consistency. This trend likely occurs because the majority of pharmacists acquire their professional knowledge and technical skills through long-term dispensing practice [59]. Due to insufficient accumulation of professional competence, younger pharmacists often struggle to secure full trust from physicians and patients [59]. Conversely, older pharmacists possess more extensive clinical experience to facilitate effective collaboration. Notably, physicians aged over 55 years and those younger than 25 years showed greater trust in clinical pharmacists than physicians aged 25–54 years. This difference may reflect age-related variation in physicians’ clinical needs. Older physicians may better recognize pharmacists’ professional value because of their extensive clinical experience [56]. Meanwhile, younger physicians rely more heavily on pharmacists’ expertise due to their own limited prescribing experience.

Optimizing compensation systems may help hospitals reduce age-related differences in exchange characteristics among clinical pharmacists. One potential approach is to increase the proportion of performance-based compensation. For example, fees for newly introduced pharmaceutical services, such as pharmacist outpatient services and medication monitoring, could be partially converted into performance-based income for pharmacists. These services were included in the 2023 National Technical Specifications for Medical Service Items issued by the National Health Commission. Linking pharmacists’ income more closely to their workload could better reward greater contributions and may, to some extent, enhance perceptions of pay Fairness. However, the actual effectiveness of these measures requires further empirical evaluation. In addition, strengthening systematic training for younger pharmacists represents another promising approach. Drawing on the experience of U.S. healthcare institutions [60,61,62], hospitals could establish mentorship programs in which experienced physicians and pharmacists provide guidance to younger pharmacists. Such programs may accelerate the development of professional knowledge while strengthening team collaboration ability through interprofessional collaborative practice, thereby improving the overall quality of healthcare services.

### 4.2. Strengths and Limitations

Exchange characteristics are the most influential factors affecting clinical interdisciplinary collaboration between pharmacists and physicians. This study is the first to examine the current status of collaboration between clinical pharmacists and physicians from the perspective of exchange characteristics, making it somewhat innovative. Additionally, the study covers most provinces across the country and has a large sample size with good representativeness.

This study has several limitations. First, a stratified multistage nonrandom sampling strategy was used, and physicians were recruited through referrals from clinical pharmacists with whom they had collaborated. Although this approach may have introduced selection bias and excluded healthcare professionals from institutions without established collaborative practices, the target population comprised healthcare professionals with collaboration experience. Therefore, the findings remain applicable to the intended study population. In addition, the exclusion of a large number of low-quality questionnaires during questionnaire screening and matching may have resulted in an overestimation of exchange characteristic levels. Second, although the Bonferroni correction was applied to control the risk of type I error arising from multiple comparisons, false-positive findings may still have occurred because of the large number of subgroup analyses. Moreover, some subgroups had relatively small sample sizes, resulting in insufficient statistical power and an increased risk of type II error. These findings therefore require further validation in studies with larger samples. Furthermore, the data had a dyadic pairing structure between clinical pharmacists and physicians. This structure may have introduced matching errors and may also have generated hospital-level clustering effects. Future studies could use multilevel models or other appropriate methods to account for these effects. Finally, although the questionnaire underwent expert consultation and pilot testing, some scales have not yet been validated in large samples of Chinese physicians and clinical pharmacists. Future studies could triangulate questionnaire findings with objective indicators, such as prescription review records and the number of clinical pharmacist interventions, to enhance the credibility of the conclusions.

## 5. Conclusions

This study examines the exchange characteristics between clinical pharmacists and physicians in interprofessional collaboration, highlighting differences across groups. Results show younger clinical pharmacists earn less physician trust and have weaker team consistency; physicians in China’s central region experience higher job burnout; and pharmacists with university degrees and higher education demonstrate better exchange traits like respect and trust. To address these issues, healthcare institutions should optimize staffing and reform compensation for fairness, universities should collaborate with hospitals on joint training, and the government should clarify clinical pharmacists’ roles and improve resource allocation to reduce regional disparities. This study represents the first empirical exploration of this topic in China. Future studies could further strengthen the content validity of the questionnaire through expert review. Building on this work, future research could also investigate causal determinants in greater depth to clarify the factors influencing exchange characteristics in clinical interprofessional collaboration and the mechanisms underlying these relationships.

## Figures and Tables

**Table 1:** Respondents and Response Rates by Income Group.

Regional Classification	No. of Hospitals (Respondents)		Response Rate (%)	
	Clinical Pharmacists	Physicians	Clinical Pharmacists	Physicians
High-income group	112 (191)	114 (176)	48.48%	45.24%
Middle-income group	84 (135)	84 (141)	44.26%	47.96%
Low-income group	78 (122)	78 (129)	46.74%	47.78%

**Table 2:** (**a**) Components of Exchange Characteristics. (**b**) Example Items in Questionnaire.

(**a**)		
**Exchange Characteristics**	**Connotations**	
Respect	An indicator of whether clinical pharmacists and physicians can freely and directly propose or accept feedback in collaboration, and the independence of their work is guaranteed.	
Trust	An indicator of a clinical pharmacist’s ability to gain the trust of a physician in his or her professional competence.	
Role Recognition	An indicator of whether clinical pharmacists and physicians can fully recognize the professional responsibilities, limitations and boundaries of both parties to avoid role conflict and identity dilution.	
Fairness	An indicator of whether clinical pharmacists and physicians believe that his effort is matched by his return	
Attitudes to collaboration		
Job Burnout	An indicator of whether clinical pharmacists and physicians can maintain stable and lasting career interest and avoid job burnout or lack of achievement	
Usefulness Perception	An indicator of whether clinical pharmacists and physicians can recognize the value of collaboration in reducing clinical burden, improving treatment quality, and reducing costs	
collaborative expectations	An indicator of whether clinical pharmacists and physicians focus on the shared responsibility of collaboration, and uphold the belief that collaborative practice will improve patient care.	
Leadership		
power influences	An indicator of whether clinical pharmacists or physicians have a higher rank in the team, assuming leadership and participating in the work assessment of the other side	
non-power influences	An indicator of whether clinical pharmacists or physicians are attractive in terms of good moral feeling or intellectual ability	
Familiarity		
team consistency	An indicator of whether clinical pharmacists and physicians can maintain consistent work schedules to improve team consistency and avoid fragmentation of patient care.	
frequency of collaboration	An indicator measures the frequency with which physicians and clinical pharmacists engage in coordinated care activities for the same patient within a defined clinical context, reflecting the operational intensity of interprofessional collaboration.	
outside of work socialization	An indicator of whether clinical pharmacists and physicians can form a good personal level of social contact.	
Communication		
team communication ability	An indicator of clinical pharmacists’ ability to communicate consistently and effectively with physicians about the treatment of patients.	
general language skills	An indicator of whether clinical pharmacists can master clinical communication skills and understand physicians’ communication preferences.	
(**b**)		
**Exchange Characteristics**	**Example Item**	
Respect	I’m respected by collaborating physicians/clinical pharmacists.	□ Disagree strongly □ Disagree a little □ Unsure □ Agree a little □ Agree strongly
Trust	For the physicians/clinical pharmacists I work with: I can rely on him/her to do a major part of the team’s work.	□ Disagree strongly □ Disagree a little □ Unsure □ Agree a little □ Agree strongly
Role Recognition	I know what my and physicians’/clinical pharmacists’ responsibilities and rights are.	□ Disagree strongly □ Disagree a little □ Unsure □ Agree a little □ Agree strongly
Fairness	In collaborative work, my contribution is proportional to the feedback from the physician/clinical pharmacist.	□ Disagree strongly □ Disagree a little □ Unsure □ Agree a little □ Agree strongly
attitudes to collaboration		
Job burnout (Reverse scoring)	Since I started this job, I have become less and less interested in my work	□ Never □ Several times a year □ Once a month □ Several times a month □ Once a week □ Several times a week □ Every day
usefulness perception	I think it’s useful for clinical pharmacists to work with physicians	□ Never □ Several times a year □ Once a month □ Several times a month □ Once a week □ Several times a week □ Every day
collaborative expectations	Physicians/clinical pharmacists and I always adhere to the patient-centered, to improve the level of rational drug use as the expectation of cooperation	□ Disagree strongly □ Disagree a little □ Unsure □ Agree a little □ Agree strongly
leadership		
power influences	Are you on the same administrative rank as a physician/clinical pharmacist?	□ Higher Rank of Physician □ Same Office Rank □ Higher Rank of Clinical Pharmacist
non-power influences	What do you think is the most attractive non-power influence for a physician/clinical pharmacist to work with? (Multiple options)	□ Moral character (morality, conduct, work style, value orientation, etc.) □ Knowledge (advanced thinking, talent, knowledge, etc.) □ Ability (organization, management, innovation, interpersonal skills, etc.) □ Affection (kindness, care, love, respect, etc.)
Familiarity		
team consistency	How would you characterize your familiarity with collaborating physicians/clinical pharmacists.	□ Strongly unfamiliar □ Somewhat unfamiliar □ Moderately familiar □ Reasonably familiar □ Highly familiar
frequency of collaboration	How frequently have you participated in co-managing patients with physicians/clinical pharmacists?	□ Hardly ever □ Infrequently □ Moderately □ Quite often □ Very frequently
outside of work socialization	For the physicians/clinical pharmacists you work with: During holidays or after office hours, I would call or visit him/her.	□ Disagree strongly □ Disagree a little □ Unsure □ Agree a little □ Agree strongly
communication		
team communication ability	Take the initiative to communicate with the doctor in time to ensure the accuracy and rationality of the doctor’s orders	□ Very bad □ Bad □ Unsure □ Good □ Very Good
general language skills	Greet the others when I see them	□ Very bad □ Bad □ Unsure □ Good □ Very Good

**Table 3:** (**a**) Basic information of sample hospitals. (**b**) Basic information of sample clinical pharmacists and physicians.

(**a**)		
**Category**		**N (%)**
Grade of hospital	Tertiary hospital	163 (59.27%)
	Secondary hospital	112 (40.73%)
Location	Eastern region	121 (44.00%)
	Central region	69 (25.09%)
	Western region	85 (30.90%)
Type of hospital	General	252 (91.63%)
	Specialized	23 (8.36%)
(**b**)		
	**Clinical Pharmacists (N(%)/Mean ± SD)**	**Physicians (N(%)/Mean ± SD)**
Gender		
Male	185 (41.29%)	226 (50.67%)
Female	263 (58.71%)	220 (49.33%)
Age	38.46 ± 7.25	42.23 ± 7.88
~25	10 (2.23%)	6 (1.35%)
25~34	161 (35.94%)	82 (18.39%)
35~44	198 (44.20%)	211 (47.31%)
45~54	72 (16.07%)	127 (28.48%)
55~	7 (1.56%)	20 (4.48%)
Marital status		
Married	383 (85.49%)	402 (90.13%)
Unmarried	55 (12.28%)	39 (8.74%)
Other (divorced, widowed, etc.)	10 (2.23%)	5 (1.12%)
years of experience	10.88 ± 6.67	15.22 ± 7.61
~5	103 (22.99%)	40 (8.97%)
6~10	159 (35.49%)	101 (22.65%)
10~20	147 (32.81%)	204 (45.75%)
20~	39 (8.71%)	101 (22.65%)
Area of Practice ^1^		
General Department	136 (30.36%)	41 (9.19%)
Internal Medicine Department	171 (38.17%)	121 (27.13%)
Surgery Department	49 (10.94%)	66 (14.80%)
Pediatrics Department	51 (11.38%)	46 (10.31%)
Gynecology Department	36 (8.04%)	43 (9.64%)
Emergency and Critical Care Department	65 (14.51%)	46 (10.31%)
Others	95 (21.21%)	107 (23.99%)
educational background		
Junior College Degree or below	18 (4.02%)	9 (2.02%)
Bachelor’s degree	214 (47.77%)	180 (40.36%)
Master’s degree	192 (42.86%)	200 (44.84%)
Doctoral Degree or above	24 (5.36%)	57 (12.78%)
Type of Employment ^2^		
Regular Employee	437 (97.54%)	437 (97.98%)
Non-regular Employee	11 (2.46%)	9 (2.02%)

Note: ^1^ Since a clinical pharmacist/physician may provide clinical services in more than one department, the percentage of the “area of practice” adds up to more than 100%. ^2^ Regular Employee include permanent staff, contract-based; Non-regular Employee include intern or temporary worker, personnel agency, others.

**Table 4:** Evaluation Results and Between-Group Comparisons of Exchange Characteristics Between Clinical Pharmacists and Physicians.

Exchange Characteristics	Clinical Pharmacists		Physicians		*t*-Test (*p*-Value)	Cohen d	P_GEE_	MD (95%Cl)
	Score	ICC (95%Cl)	Score	ICC (95%Cl)				
Respect	4.00 (±0.69)	0.60 (0.52, 0.69)	4.42 (±0.55)	0.52 (0.50, 0.55)	13.78 (<0.001)	0.847	<0.001	0.42 (0.36, 0.48)
Trust	4.32 (±0.62)	0.45 (0.38, 0.52)	4.30 (±0.52)	0.48 (0.45, 0.51)	−0.73 (0.465)	0.811	0.465	−0.02 (−0.08, 0.04)
Role recognition	4.26 (±0.53)	0.42 (0.36, 0.48)	4.36 (±0.53)	0.50 (0.47, 0.53)	3.60 (<0.001)	0.708	<0.001	0.09 (0.04, 0.14)
Fairness	3.85 (±0.68)	0.39 (0.28, 0.49)	3.98 (±0.64)	0.48 (0.38, 0.57)	4.22 (<0.001)	0.915	<0.001	0.14 (0.07, 0.20)
Job burnout (Reverse scoring)	1.74 (±0.90)	0.54 (0.46, 0.63)	1.68 (±0.93)	0.69 (0.62, 0.75)	−1.49 (0.136)	1.300	0.134	−0.07 (−0.16, 0.02)
usefulness perception	5.21 (±1.84)	0.45 (0.35, 0.54)	5.30 (±1.76)	0.44 (0.30, 0.49)	0.26 (0.343)	2.502	0.351	0.08 (−0.09, 0.26)
collaborative expectations	6.28 (±0.79)	0.45 (0.35, 0.54)	6.30 (±0.92)	0.29 (0.20, 0.35)	0.52 (0.602)	1.206	0.603	0.02 (−0.06, 0.11)
team consistency	3.95 (±0.68)	0.44 (0.34, 0.53)	3.90 (±0.75)	0.39 (0.28, 0.49)	−1.51 (0.132)	0.953	0.134	−0.05 (−0.12, 0.02)
frequency of collaboration	3.66 (±0.77)	0.41 (0.31, 0.52)	3.64 (±0.81)	0.47 (0.36, 0.58)	−0.46 (0.646)	1.096	0.652	−0.02 (−0.10, 0.06)
outside of work socialization	3.21 (±0.91)	0.61 (0.53, 0.69)	3.19 (±0.90)	0.62 (0.55, 0.70)	−0.36 (0.720)	1.263	0.723	−0.01 (−0.11, 0.07)
team communication ability	4.25 (±0.56)	0.55 (0.46, 0.64)	4.35 (±0.57)	0.57 (0.48, 0.66)	3.49 (<0.001)	0.790	<0.001	0.10 (0.04, 0.15)
general language skills	4.22 (±0.56)	0.49 (0.39, 0.58)	4.28 (±0.56)	0.53 (0.44, 0.62)	2.27 (0.023)	0.762	0.028	0.06 (0.01, 0.12)

## Data Availability

The original contributions presented in this study are included in the article. Further inquiries can be directed to the corresponding authors due to privacy concerns regarding participant confidentiality.

## Supplementary Materials

The following supporting information can be downloaded at: https://www.mdpi.com/article/10.3390/healthcare14182904/s1, Table S1. Differences in Exchange Characteristics Among Physicians Across Hospital Grades. Table S2. Differences in Exchange Characteristics Among Clinical Pharmacists Across Hospital Grades. Table S3. Differences in Exchange Characteristics Among Physicians Across Employment Types. Table S4. Differences in Exchange Characteristics Among Clinical Pharmacists Across Employment Types. Table S5. Differences in Exchange Characteristics Among Clinical Pharmacists Across Genders. Table S6. Differences in Exchange Characteristics Among Physicians Across Genders. Table S7. Differences in Exchange Characteristics Among Physicians Across Locations. Table S8. Differences in Exchange Characteristics Among Clinical Pharmacists Across Training Patterns. Table S9. Differences in Exchange Characteristics Among Clinical Pharmacists Across Ages. Table S10. Differences in Exchange Characteristics Among Physicians Across Ages. Table S11. Differences in Exchange Characteristics Among Clinical Pharmacists Across Educational Backgrounds. Table S12. Differences in Exchange Characteristics Among Physicians Across Educational Backgrounds. Table S13. Differences in Exchange Characteristics in Power Influences Between Physicians and Clinical Pharmacists. Table S14. Differences in Exchange Characteristics in Non-Power Influences Between Physicians and Clinical Pharmacists. Table S15. Reliability, collinearity, correlation analysis of the sample data of clinical pharmacists. Table S16. Reliability, collinearity, correlation analysis of the sample data of physician. Table S17. CR, and AVE of Exchange Characteristics in Clinical Pharmacist and Physician Groups. Table S18. Measurement Invariance Testing Results of Exchange Characteristics Across Clinical Pharmacist and Physician Groups.
