# Supplementary material for: Exchange Characteristics in Interprofessional Collaboration Between Clinical Pharmacists and Physicians: A National Survey
Source: Healthcare (Basel). 2026 Sep 8;14(18):2904. doi: 10.3390/healthcare14182904 (PMC13606039; doi:10.3390/healthcare14182904)
Supplement: Supplementary file 1 [file healthcare-14-02904-s001.zip › healthcare-4463805-supplementary.pdf]

Supplement S1 Subgroup Analysis Results

Table S1 Differences in Exchange Characteristics Among Physicians Across Hospital Grades

Exchange characteristics		Grade of hospital(Sample size) Tertiary Hospital(245)	Secondary Hospital(201)	P-Value	P_{GEE}	Cohen d	MD（95%CI）
Respect		4.32(±0.52)	4.25(±0.55)	0.160	0.238	0.533	-0.07(-0.17, 0.03)
Trust		4.43(±0.56)	4.38(±0.56)	0.365	0.425	0.561	-0.05(-0.15, 0.06)
Role recognition		4.36(±0.52)	4.31(±0.56)	0.331	0.408	0.539	-0.05(-0.15, 0.05)
Attitudes to collaboration	Job burnout	1.67(±0.89)	1.76(±1.06)	0.312	0.429	0.968	0.09(-0.09, 0.27)
	Usefulness perception	5.24(±1.76)	5.21(±1.85)	0.852	0.871	1.798	-0.03(-0.37, 0.31)
	collaborative expectations	6.25(±0.98)	6.32(±0.79)	0.446	0.485	0.900	0.07(-0.10, 0.23)
	team communication ability	4.34(±0.62)	4.33(±0.56)	0.880	0.898	0.593	-0.01(-0.12, 0.10)
	general language skills	4.28(±0.59)	4.27(±0.56)	0.919	0.931	0.575	-0.01(-0.11, 0.10)
fairness		3.96(±0.65)	3.96(±0.64)	0.999	0.999	0.647	0.00(-0.12, 0.12)
Familiarity	team consistency	3.88(±0.71)	3.89(±0.71)	0.895	0.907	0.709	0.01(-0.12, 0.14)
	frequency of collaboration	3.62(±0.83)	3.61(±0.84)	0.956	0.962	0.835	-0.00(-0.16, 0.15)
	outside of work socialization	3.13(±0.95)	3.19(±0.85)	0.445	0.520	0.950	0.07(-0.10, 0.24)

Table S2 Differences in Exchange Characteristics Among Clinical Pharmacists Across Hospital Grades

Exchange characteristics	Grade of hospital(Sample size) Tertiary Hospital(253)	Secondary Hospital(195)	P-Value	P_{GEE}	Cohen d	MD（95%CI）
Respect	3.98(±0.69)	4.01(±0.69)	0.614	0.772	0.694	-0.03(-0.16, 0.10)

Trust		4.32(±0.59)	4.32(±0.64)	0.960	0.850	0.621	0.00(-0.11, 0.12)
Role recognition		4.24(±0.56)	4.29(±0.52)	0.334	0.360	0.537	-0.05(-0.15, 0.05)
Attitudes to collaboration	Job burnout	1.78(±0.97)	1.74(±0.87)	0.653	0.655	0.918	0.04(-0.13, 0.21)
	Usefulness perception	5.10(±1.82)	5.31(±1.82)	0.242	0.293	1.820	-0.21(-0.55, 0.14)
	collaborative expectations	6.21(±0.82)	6.31(±0.76)	0.204	0.225	0.787	-0.10(-0.24, 0.05)
	team communication ability	4.22(±0.56)	4.25(±0.57)	0.573	0.747	0.564	-0.03(-0.14, 0.07)
	general language skills	4.21(±0.55)	4.23(±0.56)	0.764	0.903	0.559	-0.02(-0.12, 0.09)
fairness		3.84(±0.66)	3.83(±0.66)	0.902	0.842	0.682	0.01(-0.12, 0.14)
Familiarity	team consistency	3.92(±0.66)	3.93(±0.70)	0.876	0.701	0.679	0.01(-0.12, 0.14)
	frequency of collaboration	3.58(±0.74)	3.66(±0.76)	0.252	0.249	0.772	-0.08(-0.23, 0.07)
	outside of work socialization	3.27(±0.88)	3.11(±0.94)	0.075	0179	0.917	0.16(-0.01, 0.33)

Table S3 Differences in Exchange Characteristics Among Physicians Across Employment Types

**Exchange characteristics**		**Type of Employment(Sample size)**		**P-Value**	**P_{GEE}**	**Cohen d**	**MD(95%Cl)**
		**Regular Employee(437)**	**Non-regular Employee(9)**				
Respect		4.28(±0.53)	4.44(±0.46)	0.363	0.276	0.533	-0.16 (-0.52, 0.19)
Trust		4.40(±0.56)	4.53(±0.51)	0.511	0.439	0.562	-0.13 (-0.50, 0.25)
Role recognition		4.34(±0.54)	4.31(±0.46)	0.842	0.812	0.539	0.04 (-0.32, 0.39)
Attitudes to collaboration	Job burnout	1.69(±0.92)	2.89(±2.18)	<0.001	0.079	0.954	-1.20 (-1.83, -0.57)
	Usefulness perception	5.20(±1.80)	6.56(±0.73)	0.030	<0.001	1.788	-1.36 (-2.54, -0.17)
	collaborative expectations	6.28(±0.91)	6.39(±0.55)	0.723	0.545	0.900	-0.11 (-0.70, 0.49)
Communication	team communication ability	4.33(±0.59)	4.50(±0.61)	0.408	0.398	0.593	-0.17 (-0.56, 0.23)
	general language skills	4.27(±0.58)	4.42(±0.52)	0.453	0.379	0.574	-0.15 (-0.53, 0.23)
fairness		3.96(±0.65)	3.89(±0.75)	0.732	0.750	0.647	0.07 (-0.35, 0.50)

Familiarity	team consistency	3.89(±0.71)	3.78(±0.67)	0.637	0.603	0.709	0.11 (-0.36, 0.58)
	frequency of collaboration	3.62(±0.84)	3.56(±0.53)	0.830	0.721	0.838	0.06 (-0.49, 0.61)
	outside of work socialization	3.16(±0.90)	2.98(±1.19)	0.553	0.622	0.904	0.18 (-0.42, 0.78)

Table S4 Differences in Exchange Characteristics Among Clinical Pharmacists Across Employment Types

Exchange characteristics		Type of Employment(Sample size)		P-Value	P_{GEE}	Cohen d	MD(95%CI)
		Regular Employee(437)	Non-regular Employee(11)				
Respect		4.00(±0.70)	3.75(±0.45)	0.231	0.573	0.693	0.25(-0.16,0.67)
Trust		4.33(±0.62)	4.27(±0.45)	0.783	0.287	0.620	0.05(-0.32,0.42)
Role recognition		4.27(±0.53)	3.93(±0.48)	0.040	0.143	0.535	0.34(0.02,0.67)
Attitudes to collaboration	Job burnout	1.75(±0.91)	2.18(±1.08)	0.122	0.409	0.916	-0.43(-0.98,0.12)
	Usefulness perception	5.23(±1.83)	4.27(±1.62)	0.374	0.676	1.822	-0.43(-0.59,0.15)
	collaborative expectations	6.27(±0.79)	6.09(±0.66)	0.458	0.436	0.788	0.18(-0.21,0.55)
Communication	team communication ability	4.25(±0.57)	3.93(±0.25)	0.010	0.081	0.562	0.32(-0.19,0.65)
	general language skills	4.22(±0.56)	4.26(±0.43)	0.743	0.341	0.559	-0.05(-0.38,0.29)
fairness		3.85(±0.67)	3.30(±0.68)	<0.001	0.026	0.677	0.55(0.14,0.95)
Familiarity	team consistency	3.93(±0.68)	3.82(±0.60)	0.551	0.579	0.679	0.11(-0.29,0.52)
	frequency of collaboration	3.63(±0.77)	3.64(±0.81)	0.973	0.831	0.773	-0.01(-0.47,0.45)
	outside of work socialization	3.18(±0.92)	3.21(±0.70)	0.858	0.807	0.921	-0.04(-0.59,0.51)

Table S5 Differences in Exchange Characteristics Among Clinical Pharmacists Across Genders

Exchange characteristics		Gender(Sample size)		P-Value	P_{GEE}	Cohen d	MD(95%Cl)
		Male(185)	Female(263)				
Respect		4.06±0.61	3.95±0.74	0.082	0.472	0.692	0.11(-0.02,0.25)
Trust		4.36±0.56	4.30±0.66	0.280	0.517	0.619	0.06(-0.05,0.18)
Role recognition		4.29±0.48	4.25±0.57	0.541	0.436	0.538	0.03(-0.07,0.13)
Attitudes to collaboration	Job burnout	1.73±0.96	1.78±0.89	0.552	0.658	0.918	-0.05(-0.23,0.12)
	Usefulness perception	5.29±1.74	5.17±1.88	0.478	0.754	1.822	0.12(-0.22,0.47)
	Collaborative Expectations	6.32±0.69	6.22±0.85	0.180	0.037	0.787	0.10(-0.05,0.25)
Communication	Team communication Ability	4.27±0.55	4.22±0.58	0.388	0.517	0.563	0.05(-0.06,0.15)
	General language skills	4.23±0.52	4.21±0.58	0.602	0.416	0.559	0.03(-0.08,0.13)
Fairness		3.90±0.60	3.79±0.73	0.080	0.110	0.679	0.12(-0.01,0.24)
Familiarity	Team consistency	3.98±0.64	3.89±0.70	0.191	0.056	0.678	0.08(-0.04,0.21)
	Frequency of Collaboration	3.68±0.74	3.59±0.80	0.223	0.322	0.772	0.09(-0.05,0.24)
	**Outside of work Socialization**	3.28±0.91	3.11±0.92	0.064	0.268	0.917	0.17(-0.01,0.34)

Table S6 Differences in Exchange Characteristics Among Physicians Across Genders

Exchange characteristics	Gender(Sample size)		P-Value	P_{GEE}	Cohen d	MD(95%Cl)
	Male(226)	Female(220)				
**Respect**	4.23±0.58	4.34±0.48	0.040	0.038	0.531	-0.11(-0.20,-0.01)
Trust	4.38±0.59	4.43±0.53	0.402	0.380	0.561	-0.04(-0.15,0.06)
role recognition	4.32±0.57	4.36±0.51	0.381	0.381	0.539	-0.05(-0.15,0.06)

Attitudes to collaboration	Job burnout	1.72±1.05	1.71±0.88	0.900	0.893	0.969	0.01(-0.17,0.19)
	usefulness perception	5.2±1.74	5.25±1.86	0.792	0.786	1.798	-0.05(-0.38,0.29)
	collaborative expectations	6.26±0.94	6.31±0.85	0.567	0.565	0.900	-0.05(-0.22,0.12)
Communication	team communication ability	4.32±0.61	4.35±0.58	0.560	0.553	0.593	-0.03(-0.14,0.08)
	general language skills	4.26±0.6	4.29±0.54	0.581	0.579	0.575	-0.03(-0.14,0.08)
fairness		3.93±0.66	4±0.63	0.280	0.303	0.646	-0.07(-0.19,0.05)
Familiarity	team consistency	3.88±0.7	3.9±0.72	0.767	0.768	0.709	-0.02(-0.15,0.11)
	frequency of collaboration	3.61±0.82	3.62±0.85	0.842	0.824	0.838	-0.02(-0.17,0.14)
	outside of work socialization	3.14±0.93	3.18±0.88	0.670	0.667	0.907	-0.04(-0.21,0.13)

Table S7 Differences in Exchange Characteristics Among Physicians Across Locations

Exchange characteristics		Location(Sample size)			P-Value	P-Value $_{adj}$	P_{GEE}	eta^2
		Eastern Region(186)	Central Region(110)	Western Region(150)				
Respect		4.28(±0.47)	4.31(±0.60)	4.27(±0.56)	0.703	1.000	0.868	0.0016
Trust		4.37(±0.58)	4.48(±0.55)	4.39(±0.56)	0.231	0.713	0.333	0.0065
Role recognition		4.30(±0.53)	4.36(±0.54)	4.38(±0.55)	0.291	0.873	0.495	0.0056
Attitudes to collaboration	Job burnout	1.61(±0.74)	2.05(±1.37)	1.59(±0.80)	0.038	0.114	0.038	0.0147
	Usefulness perception	5.03(±1.87)	5.33(±1.62)	5.39(±1.82)	0.160	0.480	0.242	0.0082
	collaborative expectations	6.27(±0.92)	6.27(±1.02)	6.31(±0.78)	0.814	1.000	0.928	0.0010
Communication	team communication ability	4.33(±0.63)	4.40(±0.60)	4.30(±0.54)	0.250	0.750	0.496	0.0063

	general language skills	4.23(±0.57)	4.36(±0.59)	4.27(±0.57)	0.096	0.288	0.279	0.0103
	fairness	3.97(±0.62)	3.96(±0.67)	3.95(±0.66)	0.971	1.000	0.979	0.0001
	team consistency	3.89(±0.68)	3.89(±0.72)	3.88(±0.73)	0.988	1.000	0.994	0.0001
Familiarity	frequency of collaboration	3.60(±0.85)	3.66(±0.78)	3.60(±0.87)	0.912	1.000	0.818	0.0004
	outside of work socialization	3.22(±0.87)	3.06(±0.99)	3.14(±0.88)	0.663	1.000	0.491	0.0035

Table S8 Differences in Exchange Characteristics Among Clinical Pharmacists Across Training Patterns

Exchange characteristics		Pattern of training(Sample size): Training after Graduation[1](282)	Training after Job Transfer[2](148)	Direct Assignment without Training[3](18)	P-Value	P-Value adj	P_{GEE}	eta^2
**Respect**		4.07(±0.65)	3.88(±0.74)	3.79(±0.86)	0.016	0.048	0.051	0.0179
**Trust**		4.38(±0.61)	4.25(±0.9)	4.14(±0.92)	0.046	0.138	0.269	0.0134
Role recognition		4.27(±0.52)	4.27(±0.54)	4.18(±0.74)	0.893	1.000	0.818	0.0005
Attitudes to collaboration	Job burnout	1.73(±0.89)	1.81(±0.98)	1.82(±0.91)	0.714	1.000	0.647	0.0016
	Usefulness perception	5.23(±1.79)	5.22(±1.86)	5.06(±1.98)	0.961	1.000	0.828	0.0002
	Collaborative expectations	6.27(±0.73)	6.27(±0.85)	6.08(±1.09)	0.680	1.000	0.609	0.0018
**Communication**	**Team communication ability**	4.29(±0.57)	4.16(±0.55)	4.11(±0.6)	0.025	0.075	0.137	0.0163
	General language skills	4.21(±0.56)	4.21(±0.57)	4.41(±0.47)	0.331	0.993	0.242	0.0050
**Fairness**		3.89(±0.62)	3.76(±0.77)	3.67(±0.77)	0.080	0.240	0.210	0.0114
Familiarity	**Team consistency**	3.89(±0.65)	4.03(±0.7)	3.78(±0.81)	0.045	0.135	0.292	0.0136
	**Frequency of**	3.63(±0.76)	3.72(±0.73)	2.83(±0.92)	<0.001	<0.001	<0.001	0.0425

	**collaboration**							
	Outside of work socialization	3.16(±0.90)	3.26(±0.94)	2.83(±0.99)	0.115	0.345	0.680	0.0096

Note: *,**,*** indicate respectively P < 0.1， P < 0.05， P < 0.01.

[1]Training after Graduation : Graduates of clinical pharmacy programs at higher education institutions who have been employed following completion of standardized training and rotations.

[2]Training after Job Transfer: Originally employed as pharmacists/physicians in healthcare institutions, later transferred to new positions after completing standardized training.

[3]Direct Assignment without Training: Directly assigned or self-trained by the organization without undergoing standardized training.

Table S9 Differences in Exchange Characteristics Among Clinical Pharmacists Across Ages

**Exchange characteristics**		**Age(Sample size)**					**P-Value**	**P-Value $_{adj}$**	**P_{GEE}**	**eta^{2}**
		**≤25(10)**	**25~34(161)**	**35~44(198)**	**45~54(72)**	**>55(7)**				
Respect		4.13±0.71	3.92±0.78	3.99±0.66	4.15±0.53	4.36±0.63	0.182	1.000	0.144	0.0139
Trust		4.43±0.53	4.30±0.68	4.30±0.62	4.40±0.50	4.68±0.43	0.407	1.000	0.700	0.0090
Role recognition		4.13±0.57	4.22±0.59	4.29±0.54	4.31±0.39	4.71±0.39	0.122	1.000	<0.001	0.0163
	**Job burnout**	1.73±0.79	1.76±0.99	1.86±0.93	1.53±0.72	1.25±0.35	0.010	0.100	0.007	0.0286
Attitudes to collaboration	Usefulness perception	5.60±1.65	5.21±1.89	5.09±1.79	5.58±1.74	4.86±1.95	0.224	1.000	0.494	0.0129
	Collaborative expectations	6.15±0.75	6.24±0.85	6.22±0.82	6.45±0.55	6.50±0.41	0.351	1.000	0.166	0.0099
**Communication**	**Team communication ability**	4.05±0.56	4.21±0.61	4.28±0.52	4.20±0.56	4.75±0.48	0.069	0.690	0.003	0.0196
	General language skills	4.33±0.46	4.20±0.61	4.22±0.54	4.19±0.49	4.71±0.43	0.230	1.000	0.020	0.0125

**Fairness**		3.63±0.84	3.73±0.74	3.85±0.67	4.07±0.49	3.95±0.59	0.022	0.220	0.015	0.0272
Familiarity	**Team consistency**	4.10±0.74	3.80±0.73	3.93±0.65	4.15±0.55	4.14±0.69	0.009	0.090	0.022	0.0317
	Frequency of collaboration	3.60±0.70	3.61±0.76	3.61±0.79	3.75±0.73	3.14±1.07	0.453	1.000	0.527	0.0082
	**Outside of work socialization**	3.64±0.95	2.99±0.94	3.23±0.89	3.42±0.83	3.09±1.39	0.011	0.110	0.023	0.0329

Table S10 Differences in Exchange Characteristics Among Physicians Across Ages

**Exchange characteristics**		**Age(Sample size)**					**P-Value**	**P-Value adj**	**P_{GEE}**	**eta2**
		**≤25(6)**	**25~34(82)**	**35~44(211)**	**45~54(127)**	**>55(20)**				
**Respect**		4.46±0.33	4.13±0.57	4.32±0.51	4.25±0.52	4.68±0.47	<0.001	<0.001	0.002	0.0440
**Trust**		4.96±0.10	4.28±0.60	4.41±0.54	4.42±0.56	4.61±0.60	0.008	0.080	<0.001	0.0306
**role recognition**		4.38±0.49	4.11±0.56	4.37±0.53	4.38±0.51	4.75±0.37	0.007	0.070	<0.001	0.0635
Attitudes to collaboration	Job burnout	1.75±0.89	2.05±1.47	1.62±0.73	1.65±0.89	1.66±0.93	0.762	1.000	0.273	0.0042
	**usefulness perception**	6.83±0.41	5.12±1.78	5.14±1.85	5.38±1.75	5.10±1.68	0.093	0.930	0.004	0.0181
	collaborative expectations	6.83±0.26	6.26±0.90	6.22±0.94	6.35±0.86	6.43±0.75	0.241	1.000	0.020	0.0125
**Communication**	**team communication ability**	4.92±0.20	4.24±0.68	4.34±0.57	4.33±0.57	4.54±0.62	0.026	0.260	0.004	0.0248
	**general language skills**	4.85±0.26	4.21±0.62	4.28±0.56	4.26±0.57	4.45±0.60	0.049	0.490	0.027	0.0215
fairness		4.17±0.46	3.87±0.65	3.95±0.69	4.00±0.56	4.18±0.70	0.208	1.000	0.061	0.0131

Familiarity	**team consistency**	4.00±0.63	3.71±0.84	3.88±0.69	3.99±0.62	4.00±0.73	0.099	0.990	0.258	0.0175
	frequency of collaboration	3.83±0.75	3.59±0.90	3.58±0.85	3.69±0.78	3.55±0.83	0.784	1.000	0.707	0.0040
	**outside of work socialization**	2.63±1.49	3.06±0.83	3.14±0.88	3.31±0.92	2.94±1.06	0.082	0.820	0.272	0.0186

Table S11 Differences in Exchange Characteristics Among Clinical Pharmacists Across Educational Backgrounds

**Exchange characteristics**		**educational background(Sample size)**							
		**Junior College Degree or below(18)**	**Bachelor Degree(214)**	**Master Degree(192)**	**Doctor Degree or above(24)**	**P-Value**	**P-Value adj**	**P_{GEE}**	**eta-squared**
	**Respect**	3.78±0.77	3.96±0.68	4.01±0.71	4.35±0.51	0.041	0.246	0.014	0.0223
	Trust	4.22±0.47	4.31±0.57	4.33±0.69	4.50±0.53	0.466	1.000	0.143	0.0079
	Role recognition	4.06±0.53	4.28±0.51	4.26±0.58	4.39±0.47	0.253	1.000	0.092	0.0120
Attitudes to collaboration	**Job burnout**	2.14±1.19	1.86±0.99	1.64±0.81	1.59±0.73	0.067	0.402	0.243	0.0197
	Usefulness perception	5.5±1.58	5.18±1.8	5.23±1.89	5.21±1.61	0.891	1.000	0.645	0.0025
	Collaborative expectations	6.14±0.85	6.21±0.86	6.33±0.72	6.33±0.58	0.514	1.000	0.293	0.0074
**Communication**	**team Communication ability**	3.99±0.55	4.21±0.55	4.29±0.59	4.32±0.46	0.092	0.552	0.067	0.0183
	General language skills	3.96±0.56	4.24±0.55	4.22±0.58	4.21±0.46	0.402	1.000	0.098	0.0091
	Fairness	3.93±0.53	3.83±0.70	3.83±0.68	3.89±0.59	0.969	1.000	0.876	0.0012
Familiarity	Team consistency	4.00±0.77	3.93±0.69	3.93±0.67	3.83±0.56	0.912	1.000	0.912	0.0022
	Frequency of collaboration	3.56±0.70	3.61±0.76	3.64±0.81	3.71±0.62	0.724	1.000	0.570	0.0047
	Outside of work socialization	3.40±0.87	3.16±0.91	3.15±0.92	3.43±0.99	0.451	1.000	0.894	0.0083

Table S12 Differences in Exchange Characteristics Among Physicians Across Educational Backgrounds

Exchange characteristics		educational background(Sample size) Junior College Degree or below(9)	Bachelor Degree(180)	Master Degree(200)	Doctor Degree or above(57)	P-Value	P-Value $_{adj}$	P_{GEE}	eta-squared
Respect		4.19±0.46	4.31±0.55	4.24±0.54	4.38±0.48	0.432	1.000	0.235	0.0086
Trust		4.25±0.43	4.46±0.53	4.34±0.58	4.48±0.60	0.237	1.000	0.125	0.0125
**Role recognition**		4.31±0.48	4.37±0.52	4.27±0.56	4.50±0.50	0.087	0.522	0.045	0.0176
Attitudes to collaboration	Job burnout	1.72±1.12	1.79±1.00	1.69±0.98	1.54±0.75	0.291	1.000	0.366	0.0112
	usefulness perception	5.00±1.87	5.27±1.85	5.17±1.82	5.33±1.54	0.545	1.000	0.867	0.0068
	collaborative expectations	6.39±0.49	6.28±0.79	6.29±0.94	6.25±1.11	0.767	1.000	0.919	0.0040
Communication	team communication ability	4.28±0.62	4.37±0.60	4.30±0.56	4.39±0.70	0.351	1.000	0.645	0.0099
	general language skills	4.35±0.46	4.33±0.60	4.22±0.54	4.30±0.60	0.262	1.000	0.314	0.0119
fairness		3.93±0.49	3.95±0.71	3.97±0.60	3.99±0.63	0.945	1.000	0.972	0.0016
Familiarity	Team consistency	3.78±0.83	3.92±0.70	3.86±0.69	3.88±0.78	0.244	1.000	0.791	0.0123
	Frequency of collaboration	3.11±1.05	3.67±0.85	3.59±0.82	3.61±0.82	0.293	1.000	0.354	0.0114
	**Outside of work socialization**	3.22±0.83	3.04±0.90	3.19±0.84	3.41±1.10	0.087	0.522	0.148	0.0175

Table S13 Differences in Exchange Characteristics in Power Influences Between Physicians and Clinical Pharmacists

Exchange characteristics：Power influences		Clinical Pharmacists	Physicians	X^2(p)	Cramer's V
Acceptance of medical interventions	<25%	57(12.72%)	49(10.99%)	0.952	0.03
	25%~50%	127(28.35%)	125(28.03%)	(0.81)	

	50%~75%	147(32.81%)	146(32.74%)		
	>75%	117(26.12%)	126(28.25%)		
Whether physicians participate in the performance evaluation of clinical pharmacists	Yes	224（50.00%）	194(43.50%)	3.040 (0.22）	0.06
	No	224（50.00%）	252(56.50%)		
Administrative rank	Higher Rank of Clinical Pharmacist	30（6.70%）	40(8.97%)	**3.796 (0.05*)**	**0.02**
	Higher Rank of Physician	210（46.88%）	187(41.93%)		
	Same Office Rank	208（46.43%）	219(49.10%)		

Note: *,**,*** indicate respectively P < 0.1， P < 0.05， P < 0.01.

Table S14 Differences in Exchange Characteristics in Non-Power Influences Between Physicians and Clinical Pharmacists

**Exchange characteristics：Non- power influences**		**Clinical Pharmacists**	**Physicians**	**X^2(p)**	**Cramer's V**
the most appealing factors influencing collaboration.:	Moral character (morality, conduct, work style, value orientation, etc.)	264（58.93%）	259(58.07%)	**7.067 (0.07*)**	**0.01**
	Knowledge (advanced thinking, talent, knowledge, etc.)	266（59.38%）	289(64.80%)		
	Ability (organization, management, innovation, interpersonal skills, etc.)	279（62.28%）	314(70.40%)		
	Affection (kindness, care, love, respect, etc.)	37（8.26%）	65(14.75%)		
Communication methods	Face-to-face communication	402（89.73%）	409(91.70%)	0.445	0.01

	Work-related telephone communication	373（83.26%）	382(85.65%)	（0.93）
	Communication and Social Networking Software	330（73.66%）	325(72.87%)	
	In-hospital electronic information system	155（34.60%）	167(37.44%)	

Note: *,**,*** indicate respectively P < 0.1，P < 0.05，P < 0.01.

Supplement S2 Questionnaire psychometric results

Table S15 Reliability, collinearity, correlation analysis of the sample data of clinical pharmacists

Variables	Pre-α	α	VIF	(1)	(2)	(3)	(4)	(5)	(6)	(7)	(8)	(9)	(10)	(11)	(12)	(13)	(14)	(15)	(16)	(17)	(18)	(19)	(20)	(21)	(22)	(23)	(24)	(25)
(1) Y	0.95	0.96	-	1.000																								
(2) X1-11-1	0.87	0.82	1.39	0.188 ***	1.000																							
(3) X1-11-2	0.21	0.03	1.14	0.135 ***	0.062	1.000																						
(4) X1-11-3	0.42	0.45	1.58	0.321 ***	0.342 ***	0.156 ***	1.000																					
(5) X1-11-4	0.66	0.46	1.64	0.206 ***	0.464 ***	0.176 ***	0.452 ***	1.000																				
(6) X1-11-5	0.37	0.34	1.18	0.207 ***	0.205 ***	0.142 ***	0.264 ***	0.213 ***	1.000																			
(7) X2-1-1	0.92	0.89	2.41	0.393 ***	0.242 ***	0.052	0.225 ***	0.200 ***	0.098 **	1.000																		
(8) X2-1-2	0.87	0.88	3.19	0.418 ***	0.205 ***	-0.007	0.196 ***	0.203 ***	0.152 ***	0.673 ***	1.000																	
(9) X2-1-3	0.84	0.85	3.11	0.398 ***	0.223 ***	0.037 ***	0.230	0.165 ***	0.170 ***	0.698 ***	0.761 ***	1.000																
(10) X2-2-1	0.97	0.82	1.44	-0.156 ***	-0.192 ***	-0.065	-0.309 ***	-0.309 ***	-0.085 *	-0.267 ***	-0.257 ***	-0.206 ***	1.000															
(11) X2-2-2	0.81	0.78	1.47	0.268 ***	0.227	0.061	0.234 ***	0.220 ***	0.061	0.349 ***	0.339 ***	0.351 ***	-0.317 ***	1.000														
(12) X2-2-3	0.71	0.87	2.08	0.233 ***	0.157 ***	0.041	0.278 ***	0.175 ***	0.090 **	0.335 ***	0.339 ***	0.290 ***	-0.399 ***	0.406 ***	1.000													
(13) X2-3-1	0.80	0.70	1.23	0.128 ***	0.068	0.047	0.232 ***	0.173 ***	0.136 ***	0.049	-0.034	-0.012	-0.202 ***	-0.011	0.114 **	1.000												
(14) X2-3-2	0.82	0.85	2.04	0.391 ***	0.217 ***	0.051	0.279 ***	0.173 ***	0.159 ***	0.467 ***	0.554 ***	0.529 ***	-0.182 ***	0.357 ***	0.290 ***	-0.022	1.000											
(15) X3-1	0.96	0.89	3.31	0.453 ***	0.228 ***	0.066	0.262 ***	0.230 ***	0.187 ***	0.586 ***	0.673 ***	0.637 ***	-0.267 ***	0.396 ***	0.394 ***	-0.018	0.665 ***	1.000										
(16) X3-2	0.97	0.93	2.31	0.394 ***	0.230 ***	0.120 ***	0.252 ***	0.232 ***	0.142 ***	0.516 ***	0.453 ***	0.517 ***	-0.233 ***	0.257 ***	0.263 ***	0.102 **	0.503 ***	0.641 ***	1.000									
(17) X3-3	0.81	0.86	2.14	0.388 ***	0.240 ***	0.161 ***	0.321 ***	0.330 ***	0.204 ***	0.463 ***	0.444 ***	0.492 ***	-0.237 ***	0.312 ***	0.257 ***	0.035	0.506 ***	0.572 ***	0.582 ***	1.000								
(18) X3-5-1	0.83	0.92	1.59	-0.270 ***	-0.209 ***	-0.180 ***	-0.367 ***	-0.291 ***	-0.181 ***	-0.318 ***	-0.271 ***	-0.289 ***	0.263 ***	-0.258 ***	-0.349 ***	-0.233 ***	-0.296 ***	-0.378 ***	-0.288 ***	-0.294 ***	1.000							
(19) X3-5-2	-	-	1.26	0.244 ***	0.095 **	0.073	0.199 ***	0.171 ***	0.125 ***	0.200 ***	0.257 ***	0.232 ***	-0.204 ***	0.192 ***	0.210 ***	0.151 ***	0.231 ***	0.234 ***	0.304 ***	0.253 ***	-0.196 ***	1.000						
(20) X3-5-3	0.80	0.74	1.80	0.380 ***	0.216 ***	0.150 ***	0.362 ***	0.251 ***	0.174 ***	0.345 ***	0.332 ***	0.362 ***	-0.182 ***	0.278 ***	0.248 ***	0.094 **	0.399 ***	0.501 ***	0.460 ***	0.413 ***	-0.491 ***	0.333 ***	1.000					
(21) X3-4	0.74	0.82	2.35	0.281 ***	0.170 ***	0.048	0.249 ***	0.204 ***	0.087 *	0.462 ***	0.457 ***	0.436 ***	-0.430 ***	0.427 ***	0.647 ***	0.051	0.364 ***	0.502 ***	0.434 ***	0.418 ***	-0.322 ***	0.247 ***	0.299 ***	1.000				
(22) X3-7-1	-	-	1.27	0.191 ***	0.085 *	0.078 *	0.161 ***	0.139 ***	0.165 ***	0.242 ***	0.262 ***	0.233 ***	-0.197 ***	0.222 ***	0.188 ***	0.072	0.204 ***	0.297 ***	0.281 ***	0.267 ***	-0.195 ***	0.230 ***	0.268 ***	0.300 ***	1.000			
(23) X3-7-2	0.90	0.90	1.40	0.202 ***	0.162 ***	-0.021	0.135 ***	0.133 ***	0.153 ***	0.252 ***	0.298 ***	0.296 ***	-0.218 ***	0.307 ***	0.331 ***	-0.098 **	0.248 ***	0.317 ***	0.205 ***	0.275 ***	-0.138 ***	0.152 ***	0.156 ***	0.352 ***	0.310 ***	1.000		
(24) X3-8-1	0.83	0.88	2.81	0.423 ***	0.264 ***	0.145 ***	0.299 ***	0.313 ***	0.161 ***	0.462 ***	0.459 ***	0.507 ***	-0.194 ***	0.291 ***	0.174 ***	0.073	0.480 ***	0.552 ***	0.587 ***	0.605 ***	-0.282 ***	0.223 ***	0.433 ***	0.397 ***	0.299 ***	0.268 ***	1.000	
(25) X3-8-2	0.95	0.92	2.50	0.414 ***	0.224 ***	0.212 ***	0.240 ***	0.233 ***	0.155 ***	0.437 ***	0.405 ***	0.478 ***	-0.187 ***	0.305 ***	0.148 ***	0.029	0.404 ***	0.475 ***	0.511 ***	0.575 ***	-0.224 ***	0.227 ***	0.366 ***	0.371 ***	0.244 ***	0.320 ***	0.727 ***	1.000

Table S16 Reliability, collinearity, correlation analysis of the sample data of physician

Variables	Pre-α	α	VIF	(1)	(2)	(3)	(4)	(5)	(6)	(7)	(8)	(9)	(10)	(11)	(12)	(13)	(14)	(15)	(16)	(17)	(18)	(19)	(20)	(21)	(22)	(23)	(24)	(25)
(1) Y	0.98	0.97	-	1.000																								
(2) X1-11-1	0.86	0.81	1.48	0.139 ***	1.000																							
(3) X1-11-2	0.06	0.04	1.14	0.120 ***	-0.009	1.000																						
(4) X1-11-3	0.66	0.38	1.53	0.156 ***	0.324 ***	0.140 ***	1.000																					
(5) X1-11-4	0.84	0.45	1.63	0.214 ***	0.488 ***	0.150 ***	0.423 ***	1.000																				
(6) X1-11-5	0.65	0.24	1.19	0.115 **	0.304 ***	0.075 *	0.240 ***	0.186 ***	1.000																			
(7) X2-1-1	0.94	0.90	3.00	0.513 ***	0.140 ***	0.127 ***	0.292 ***	0.275 ***	0.116 ***	1.000																		
(8) X2-1-2	0.89	0.88	2.80	0.488 ***	0.164 ***	0.053	0.178 ***	0.234 ***	0.098**	0.681***	1.000																	
(9) X2-1-3	0.88	0.86	2.65	0.429 ***	0.169 ***	0.034	0.249 ***	0.244 ***	0.118 ***	0.721 ***	0.667 ***	1.000																
(10) X2-2-1	0.79	0.84	1.60	-0.131 ***	-0.187 ***	-0.007	-0.140 ***	-0.232 ***	-0.096 **	-0.198 ***	-0.294 ***	-0.207 ***	1.000															
(11) X2-2-2	0.82	0.87	1.66	0.259 ***	0.208 ***	0.084 *	0.260 ***	0.251 ***	0.168 ***	0.392 ***	0.438 ***	0.389 ***	-0.410 ***	1.000														
(12) X2-2-3	0.60	0.89	2.16	0.220 ***	0.132 ***	-0.019	0.204 ***	0.193 ***	0.150 ***	0.327 ***	0.377 ***	0.342 ***	-0.454 ***	0.485 ***	1.000													
(13) X2-3-1	0.86	0.72	1.33	0.058	0.161 ***	0.094 **	0.255 ***	0.206 ***	0.124 ***	0.088 *	0.029	0.072	-0.306 ***	0.184 ***	0.097 **	1.000												
(14) X2-3-2	0.93	0.87	2.66	0.388 ***	0.153 ***	0.100 **	0.300 ***	0.267 ***	0.130 ***	0.575 ***	0.561 ***	0.606 ***	-0.208 ***	0.397 ***	0.361 ***	0.048	1.000											
(15) X3-1	0.83	0.90	3.48	0.401 ***	0.149 ***	0.073	0.312 ***	0.290 ***	0.084 *	0.661 ***	0.657 ***	0.635 ***	-0.226 ***	0.387 ***	0.352 ***	0.058	0.727 ***	1.000										
(16) X3-2	0.93	0.93	2.94	0.523 ***	0.087*	0.179 ***	0.239 ***	0.252 ***	0.102 **	0.647 ***	0.578 ***	0.563 ***	-0.152 ***	0.333 ***	0.258 ***	0.090 **	0.613 ***	0.664 ***	1.000									
(17) X3-3	0.90	0.87	2.19	0.391 ***	0.161 ***	0.104 **	0.352 ***	0.314 ***	0.098 **	0.591 ***	0.506 ***	0.533 ***	-0.182 ***	0.339 ***	0.319 ***	0.102 **	0.612 ***	0.643 ***	0.608 ***	1.000								
(18) X3-5-1	0.89	0.92	1.46	-0.133 ***	-0.171 ***	-0.114 **	-0.307 ***	-0.265 ***	-0.151 ***	-0.258 ***	-0.138 ***	-0.207 ***	0.317 ***	-0.295 ***	-0.267 ***	-0.326 ***	-0.291 ***	-0.290 ***	-0.204 ***	-0.251 ***	1.000							
(19) X3-5-2	-	-	1.31	0.373 ***	0.138 ***	0.072 ***	0.192 ***	0.164 ***	0.051	0.244 ***	0.281 ***	0.185 ***	-0.133 ***	0.202 ***	0.198 ***	0.119 ***	0.242 ***	0.254 ***	0.345 ***	0.301 ***	-0.129 ***	1.000						
(20) X3-5-3	0.96	0.86	1.57	0.390 ***	0.066	0.133 ***	0.188 ***	0.150 ***	0.046	0.358 ***	0.310 ***	0.323 ***	-0.074 *	0.139 ***	0.156 ***	0.038	0.404 ***	0.356 ***	0.481 ***	0.323 ***	-0.238 ***	0.344 ***	1.000					
(21) X3-4	0.87	0.88	2.57	0.285 ***	0.143 ***	0.070	0.167 ***	0.210 ***	0.149 ***	0.453 ***	0.504 ***	0.429 ***	-0.378 ***	0.463 ***	0.658 ***	0.028	0.518 ***	0.551 ***	0.460 ***	0.433 ***	-0.279 ***	0.270 ***	0.338 ***	1.000				
(22) X3-7-1	-	-	1.45	0.247 ***	0.069	0.061	0.173 ***	0.086 *	0.030	0.275 ***	0.306 ***	0.235 ***	-0.066	0.181 ***	0.134 ***	0.084 *	0.245 ***	0.305 ***	0.377 ***	0.242 ***	-0.167 ***	0.281 ***	0.297 ***	0.296 ***	1.000			
(23) X3-7-2	0.94	0.91	1.42	0.203 ***	0.118 ***	-0.072	0.085 *	0.116 ***	0.125 ***	0.213 ***	0.294 ***	0.252 ***	-0.217 ***	0.256 ***	0.338 ***	-0.058	0.254 ***	0.254 ***	0.246 ***	0.201 ***	-0.117 ***	0.187 ***	0.105 **	0.358 ***	0.369 ***	1.000		
(24) X3-8-1	0.94	0.92	3.36	0.483 ***	0.131 ***	0.148 ***	0.295 ***	0.309 ***	0.050	0.562 ***	0.547 ***	0.482 ***	-0.094 **	0.264 ***	0.234 ***	0.113 **	0.511 ***	0.608 ***	0.684 ***	0.547 ***	-0.247 ***	0.354 ***	0.456 ***	0.436 ***	0.419 ***	0.245 ***	1.000	
(25) X3-8-2	0.97	0.94	3.35	0.522 ***	0.130 ***	0.186 ***	0.293 ***	0.300 ***	0.096 **	0.556 ***	0.579 ***	0.519 ***	-0.119 ***	0.262 ***	0.246 ***	0.038	0.558 ***	0.647 ***	0.636 ***	0.550 ***	-0.178 ***	0.355 ***	0.466 ***	0.433***	0.383 ***	0.304 ***	0.781 ***	1.000

Table S17 CR, and AVE of Exchange Characteristics in Clinical Pharmacist and Physician Groups

Exchange characteristics	Item	Clinical Pharmacists			Physicians		
		Standardized factor loadings	CR	AVE	Standardized factor loadings	CR	AVE
**Respect**	res1	0.838	0.905	0.704	0.901	0.937	0.789
	res2	0.792			0.860		
	res3	0.855			0.896		
	res4	0.869			0.896		
**Trust**	tur1	0.923	0.941	0.798	0.788	0.904	0.701
	tur2	0.89			0.819		
	tur3	0.885			0.884		
	tur4	0.875			0.856		
**Role Recognition**	rol1	0.859	0.878	0.645	0.766	0.883	0.655
	rol2	0.86			0.759		
	rol3	0.732			0.882		
	rol4	0.752			0.825		
**Fairness**	fai1	0.963	0.866	0.690	0.671	0.867	0.688
	fai2	0.883			0.905		
	fai3	0.603			0.891		
**Job burnout**	job1	0.800	0.916	0.733	0.841	0.915	0.730
	job2	0.826			0.828		
	job3	0.900			0.875		
	job4	0.895			0.873		
**usefulness perception**	use1	-	-	-	-	-	-
**collaborative expectations**	col1	0.773	0.788	0.650	0.721	0.796	0.664

	col2	0.838			0.899		
**team consistency**	tea1	-	-	-	-	-	-
**frequency of collaboration**	fre1	-	-	-	-	-	-
	out1	0.722			0.741		
	out2	0.844			0.724		
**outside of work socialization**	out3	0.853	0.896	0.635	0.851	0.891	0.621
	out4	0.805			0.838		
	out5	0.751			0.779		
	tea1	0.819			0.889		
**team communication ability**	tea2	0.802	0.898	0.687	0.882	0.933	0.777
	tea3	0.845			0.858		
	tea4	0.848			0.897		
	gen1	0.685			0.703		
	gen2	0.759			0.773		
	gen3	0.770			0.812		
	gen4	0.835			0.866		
**general language skills**	gen5	0.883	0.938	0.628	0.858	0.945	0.659
	gen6	0.878			0.910		
	gen7	0.893			0.887		
	gen8	0.672			0.693		
	gen9	0.717			0.773		

Note: CR $\geq$ 0.70 indicates good internal consistency of the factor, and AVE $\geq$ 0.50 indicates adequate convergent validity.

Table S18 Measurement Invariance Testing Results of Exchange Characteristics Across Clinical Pharmacist and Physician Groups

Model	CFI	ΔCFI	TLI	CMIN	DF	CMIN/DF	$\Delta\chi^2$ (P)	RMSEA	ΔRMSEA
**M Clinical Pharmacists**	0.927	-	0.919	1693.95	666	2.543	-	0.059	-
**M Physicians**	0.932	-	0.924	1648.64	666	2.475	-	0.058	-
**M1 Unconstrained**	0.929	-	0.921	3342.41	1332	2.509	-	0.041	-
**M2 Measurement weights**	0.929	0.000	0.923	3381.03	1362	2.482	38.617 (0.135)	0.041	0.000
**M3 Structural covariances**	0.921	-0.008	0.917	3648.29	1406	2.593	267.712 (<0.001)	0.042	0.001
